# VIA1 is a conserved regulator of thylakoid membrane integrity that acts through VIPP1

**DOI:** 10.1073/pnas.2605816123

**Published:** 2026-08-17

**Authors:** Pamela Vetrano, Kelsey Krall, Laura Martinez, Eleonora Traverso, Tomas Morosinotto, Nicholas A. T. Irwin, Yuval Mazor, Silvia Ramundo

**Affiliations:** ^a^ Gregor Mendel Institute, Austrian Academy of Sciences, Vienna BioCenter, Vienna 1030, Austria; ^b^ Vienna BioCenter PhD Program, Doctoral School of the University of Vienna and Medical University of Vienna, Vienna 1030, Austria; ^c^Biodesign Institute, School of Molecular Sciences, Arizona State University, Tempe, AZ 85281; ^d^ Dipartimento di Biologia, Università di Padova, Padova 35131, Italy

**Keywords:** thylakoid membrane maintenance, high light acclimation, *Chlamydomonas reinhardtii*, *Synechocystis sp*. pcc 6803, VIPP1

## Abstract

Thylakoid membranes are indispensable for oxygenic photosynthesis, yet the mechanisms that protect these membranes from photooxidative damage remain poorly understood. By screening poorly characterized proteins induced during the chloroplast unfolded protein response, we identify VIA1 as an essential factor for preserving thylakoid integrity under high light in the model green alga *Chlamydomonas reinhardtii*. Loss of VIA1 causes hypersensitivity to photo-oxidative stress and rapid thylakoid swelling. VIA1 localizes to thylakoid membranes and directly binds Vesicle-Inducing Protein in Plastids 1 (VIPP1), an ESCRT-III-like protein essential for thylakoid biogenesis and remodeling. Structure-guided mutagenesis shows that this interaction is required for VIA1 function and is mediated by a winged-helix domain interface reminiscent of ESCRT-II/ESCRT-III binding mode. VIA1 orthologs from cyanobacteria and land plants rescue the Chlamydomonas *via1* mutant phenotype, and disruption of VIA1 in *Synechocystis sp*. PCC 6803 impairs growth, especially under light stress. Together, these findings establish VIA1 as an evolutionarily conserved protein that contributes to thylakoid membrane homeostasis via its interaction with VIPP1.

## Significance

All organisms performing oxygenic photosynthesis rely on thylakoid membranes to capture light and produce oxygen. Yet these membranes are highly susceptible to environmental stress, particularly excess light, which causes oxidative damage to membrane lipids and proteins. How thylakoid integrity is maintained under these conditions remains an open question. Here, we show that VIA1, a highly conserved protein, is required for maintaining thylakoid structure under high light in the green alga *Chlamydomonas reinhardtii.* VIA1 interacts with Vesicle-Inducing Protein in Plastids 1 (VIPP1), an ESCRT-III-like protein essential for thylakoid biogenesis, through a functionally indispensable interface reminiscent of ESCRT-II/ESCRT-III binding mode. The conservation of the VIA1–VIPP1 module from cyanobacteria to land plants suggests it arose early in the evolution of oxygenic photosynthesis and has been maintained ever since.

Photosynthesis, essential for much of life on Earth, is dependent on the extensive network of highly dynamic and specialized membranes known as thylakoids. This membrane system predates the origin of chloroplasts, as it is also present in cyanobacteria. While its composition has remained highly conserved through evolution (1), the spatial organization of thylakoids has undergone significant change over time (2, 3), suggesting that their biogenesis has also evolved. The complexity of this process—which involves the coordinated synthesis, assembly, and integration of lipids, proteins, and pigments—and its susceptibility to external stress stimuli, has impeded efforts to elucidate the underlying mechanisms. In fact, over the past twenty years, only a few of the molecular components directly involved in the biogenesis and maintenance of thylakoid membranes have been discovered (4–10).

One of the most extensively investigated of these factors is Vesicle Inducing Protein in Plastid 1 (VIPP1), an essential protein found in nearly all oxygenic photosynthetic organisms (11). Several studies have pointed to VIPP1’s involvement in thylakoid biogenesis (4, 11–13) as well as in chloroplast envelope and thylakoid membrane maintenance (14–17). Most notably, the deletion of the *VIPP1* gene in the land plant *Arabidopsis thaliana* and the cyanobacterium *Synechocystis sp.* PCC6803 was shown to induce severe abnormalities in their thylakoid membrane architecture (4, 11, 15). Another study in the unicellular green alga *Chlamydomonas reinhardtii* demonstrated that downregulation of VIPP1 via RNA interference causes light-induced thylakoid swelling (14). A similar phenotype was also observed in Arabidopsis chloroplasts upon knockdown of VIPP1 (15) and in Synechocystis cells expressing a mutant variant of VIPP1 (18).

Recent studies on cyanobacterial VIPP1 have revealed that this protein shares an evolutionary origin and structural features with the Endosomal Sorting Complex Required for Transport (ESCRT)-III components of eukaryotic cells (18, 19). The ESCRT machinery mediates membrane remodeling events in a wide range of cellular contexts and was long thought to be restricted to eukaryotes. However, the discovery of ESCRT-III-like proteins in archaea, cyanobacteria, and chloroplasts has uncovered an ancient, conserved membrane-remodeling module that likely predates the emergence of eukaryotic cells (18–22). Despite these advances, how VIPP1 is controlled in vivo—and whether it acts with dedicated partners to preserve thylakoids, particularly under stress—remains poorly understood.

To address this question, we leveraged the chloroplast unfolded protein response (cpUPR) to identify VIPP1-interacting proteins that contribute to thylakoid membrane integrity and photoprotection. In Chlamydomonas, cpUPR activation—triggered by artificial repression of an essential chloroplast protease, or by physiological photoxidative stress—drives a nuclear gene expression program that requires the cytosolic Ser/Thr kinase MARS1 (23). During this response, VIPP1 is induced alongside protein chaperones, proteases, and multiple poorly annotated genes, suggesting a coordinated effort to stabilize the highly protein-rich thylakoid membranes when chloroplast proteostasis is challenged. This observation prompted us to examine uncharacterized cpUPR-induced factors for their potential roles in membrane homeostasis. Among these, we focused on CPLD50, which we renamed VIPP1-Associated Protein 1 (VIA1) (24, 25). VIA1 is conserved from cyanobacteria to land plants and contains two winged-helix (WH) domains, a fold typically found in ESCRT-II proteins and known to mediate interactions with ESCRT-III proteins (24, 26).

Here, we show that *via1* hypomorphic mutants are hypersensitive to photo-oxidative stress and exhibit thylakoid membrane perturbations specifically under high light exposure, reminiscent of phenotypes reported for VIPP1 knockdown in Chlamydomonas. Mechanistically, we demonstrate that VIA1 binds VIPP1 directly via its WH domains, and that this interaction is required for VIA1 function. Moreover, the VIA1–VIPP1 module is functionally conserved, as VIA1 orthologs from Synechocystis and Arabidopsis rescue a Chlamydomonas *via1* mutant. Thus, VIA1 is a conserved regulator of VIPP1-dependent membrane remodeling.

## Results

### VIA1 (CPLD50) Is a Highly Conserved Protein Upregulated Upon Photooxidative Stress in a MARS1-Dependent Manner.

We reasoned that proteins acting alongside VIPP1 to preserve thylakoid structural integrity should be conserved across photosynthetic lineages, chloroplast-localized, and capable of associating with membranes. We therefore prioritized candidates with these characteristics among the MARS1-dependent genes activated during the cpUPR. CPLD50 (hereafter VIA1) emerged as a strong hit for three reasons: i) it belongs to the GreenCut (27), a group of nuclear-encoded proteins restricted to green-lineage organisms, consistent with a photosynthesis- or chloroplast-associated function; ii) it has been previously detected in proteomic surveys aimed at defining chloroplast-localized proteins and is also predicted to be chloroplast-targeted [TargetP2.0 (28)/WoLF PSORT (29)/DeepLoc (30)]; and iii) it is predicted to encode three transmembrane helices, suggesting membrane association.

To confirm that VIA1 expression is stress-responsive, we performed RT–qPCR analysis in wild-type (WT) and *mars1* knockout (*mars1*) cultures exposed to high light (HL) and monitored the expression of *VIA1* alongside canonical cpUPR marker genes (*SNOAL2*, *VIPP2*, *HSP22E*). In WT cells, all tested genes—including *VIA1*—were strongly induced by photo-oxidative stress, whereas this induction was abolished in the *mars1* mutant. These results confirmed that *VIA1* expression is induced by HL in a MARS1-dependent manner (*SI Appendix*, Fig. S1).

**Fig. 1.**
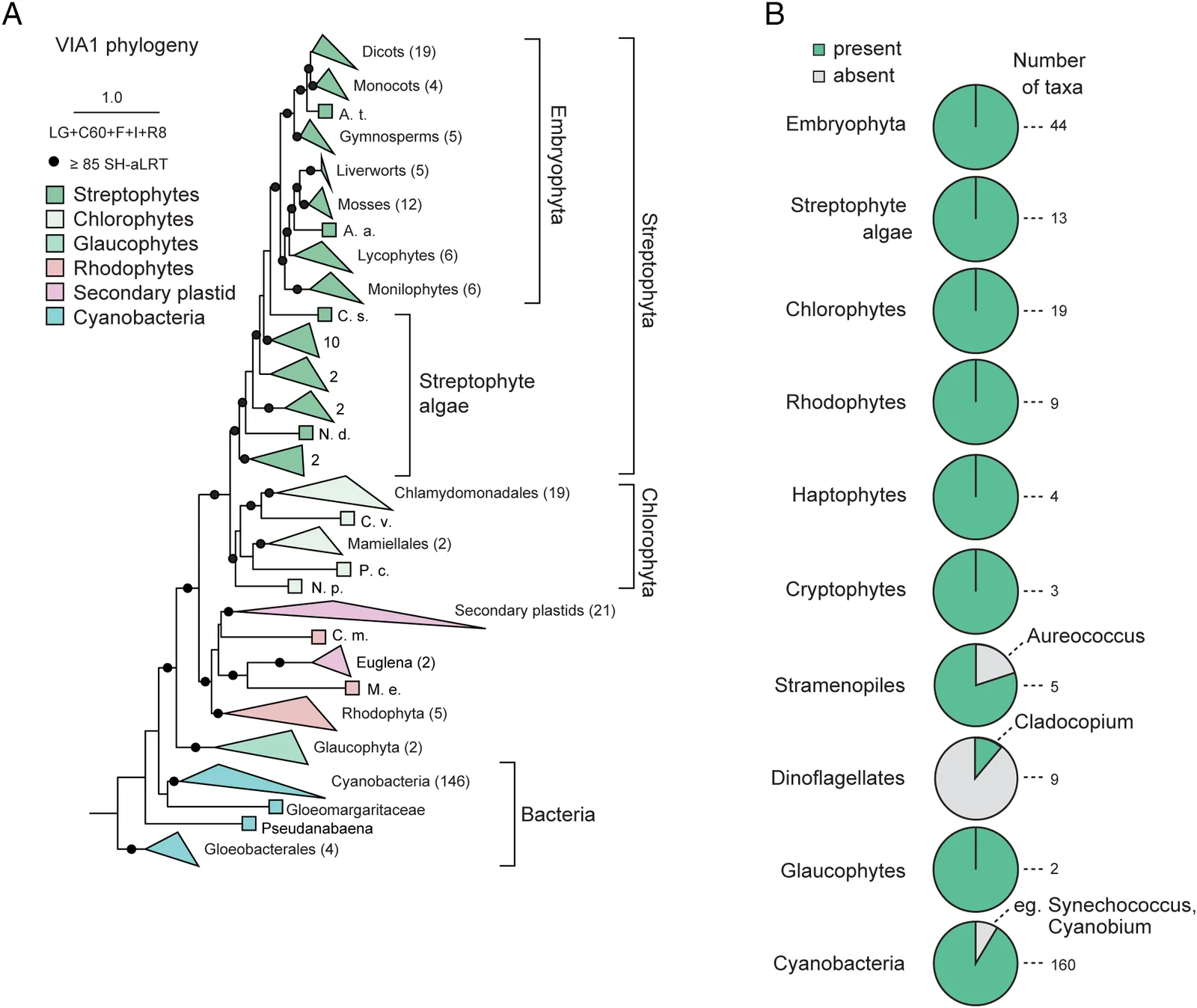
VIA1 is an evolutionarily conserved protein across photosynthetic lineages. (*A*) Maximum-likelihood phylogeny of VIA1. Taxonomic groups have been colored as noted, and triangles reflect collapsed clades with the total number of taxa denoted in parentheses. Scale bar reflects the average number of substitutions per site. SH-aLRT, Shimodaira–Hasegawa approximate likelihood ratio test. (*B*) Presence of VIA1 across key photosynthetic lineages. The full phylogeny and species distribution are available at https://itol.embl.de/shared/1WjO4fmjYiy3z.

Next, to validate and extend the Green Cut assignment, we reconstructed a VIA1 phylogeny and assessed its distribution across eukaryotic and prokaryotic lineages (Fig. 1 *A* and *B*). VIA1 is highly conserved across most photosynthetic eukaryotes, including species bearing primary and secondary plastids, but has been frequently lost in photosynthetic dinoflagellates. This distribution, together with the presence of homologs in cyanobacteria, supports a plastid-associated origin for VIA1 dating back to the primary cyanobacterial endosymbiosis in the Viridiplantae.

### Loss of VIA1 Leads to Thylakoid Structural Alterations Under High Light and Renders Chlamydomonas Hypersensitive to Photooxidative Stress.

**Fig. 2.**
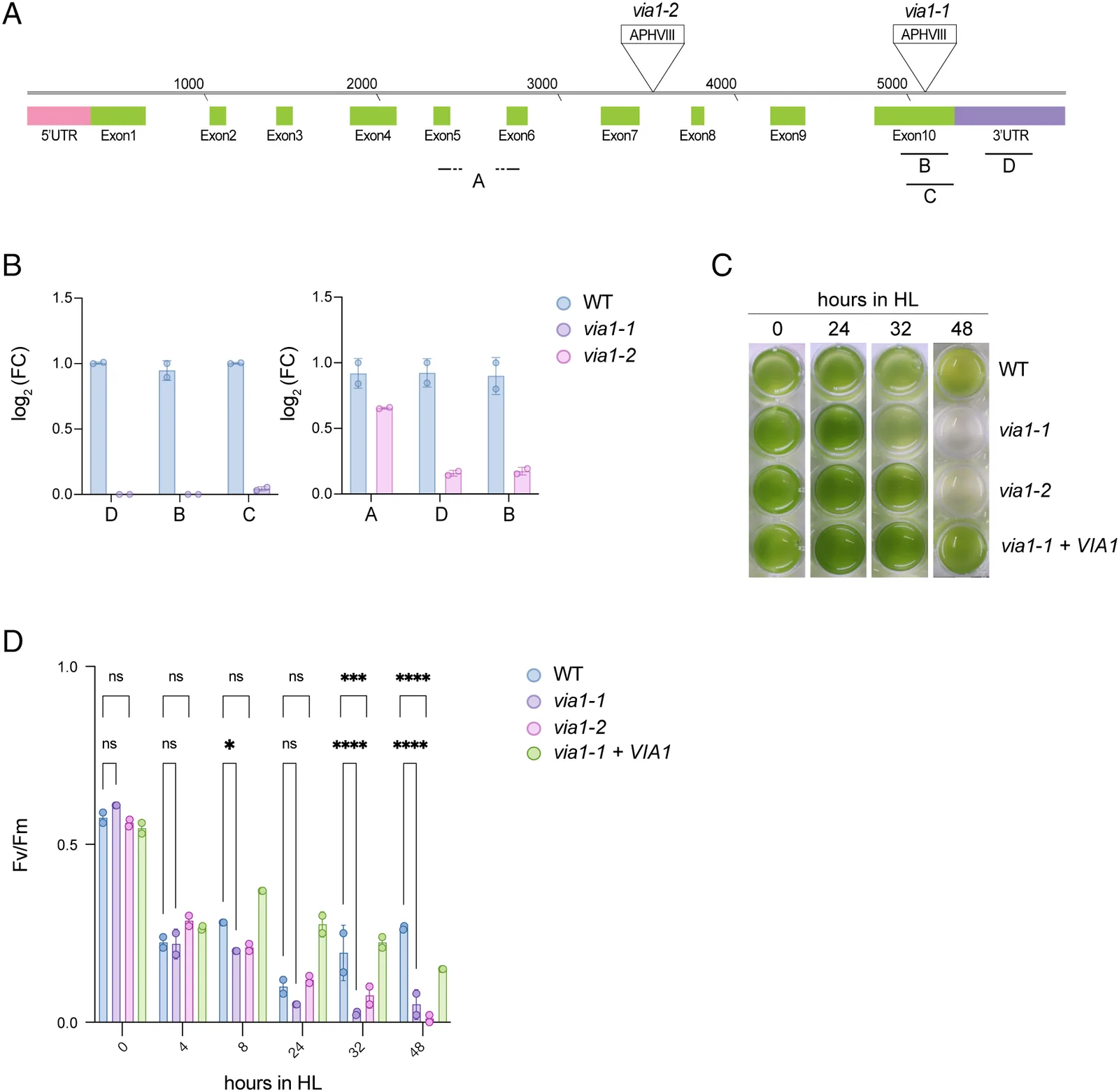
VIA1 is required for high light acclimation. (*A*) *VIA1* gene model showing exons, 5′ and 3′ untranslated regions (UTRs). The position of the mutagenic cassette carrying the paromomycin resistance gene (*APHVIII*) in *via1-1* and *via1-2* is indicated. Letters (*A*–*D*) denote the amplicon regions used for RT–qPCR in (*B*). (*B*) *VIA1* transcript levels in *via1-1* and *via1-2*, quantified by RT–qPCR using the regions indicated in (*A*). *GBLP* served as the reference gene for normalization. (*C*) Representative images of liquid TAP cultures of WT, *via1-1*, *via1-2*, and *via1-1*+*VIA1* at various time points during a 48-h HL treatment. (*D*) Photosystem II quantum yield (Fv/Fm) measured over 48 h of HL exposure in WT, *via1-1*, *via1-2*, and *via1-1*+*VIA1* strains. The significance was assessed via Two-Way ANOVA and Tukey’s multiple comparisons analysis.

To address the functional role of VIA1, we obtained two independent insertional *via1* mutant strains of *C. reinhardtii*, hereafter referred to as *via1-1* and *via1-2*, from the Chlamydomonas Library Project (CLiP) (31). The insertion sites within the *VIA1* locus were confirmed by PCR (*SI Appendix*, Fig. S2 *B*–*D*) and backcross analyses (*SI Appendix*, Fig. S2 *E* and *F*). Moreover, RT-qPCR (Fig. 2 *A* and *B*) suggested that VIA1 expression is reduced in both *via1-1* and *via1-2*.

When grown under standard light conditions, *via1-1* and *via1-2* mutants were indistinguishable from WT. This was also supported by measurements of key photosynthetic parameters, including the quantum yield of photosystem I (Y_I_), the maximum quantum efficiency of photosystem II (Fv/Fm), the plastoquinone redox state (1-qL), and nonphotochemical quenching (NPQ) (*SI Appendix*, Fig. S3 *A*–*D*), as well as by growth assays on TAP agar (*SI Appendix*, Fig. S3 *E* and *F*). In contrast, when exposed to HL, both mutants were significantly more sensitive to photooxidative stress than WT. In particular, chlorophyll content was significantly reduced in mutants compared to WT after 2 d, and photobleaching occurred earlier in the mutants (Fig. 2*C* and *SI Appendix*, Fig. S4 *A* and *B*). Consistently, Fv/Fm declined significantly in both mutants after approximately 32 h of HL exposure (Fig. 2*D*). These data rule out a primary photosynthetic defect in either mutant while demonstrating that VIA1 is essential for maintaining photosynthetic function under photooxidative stress.

**Fig. 3.**
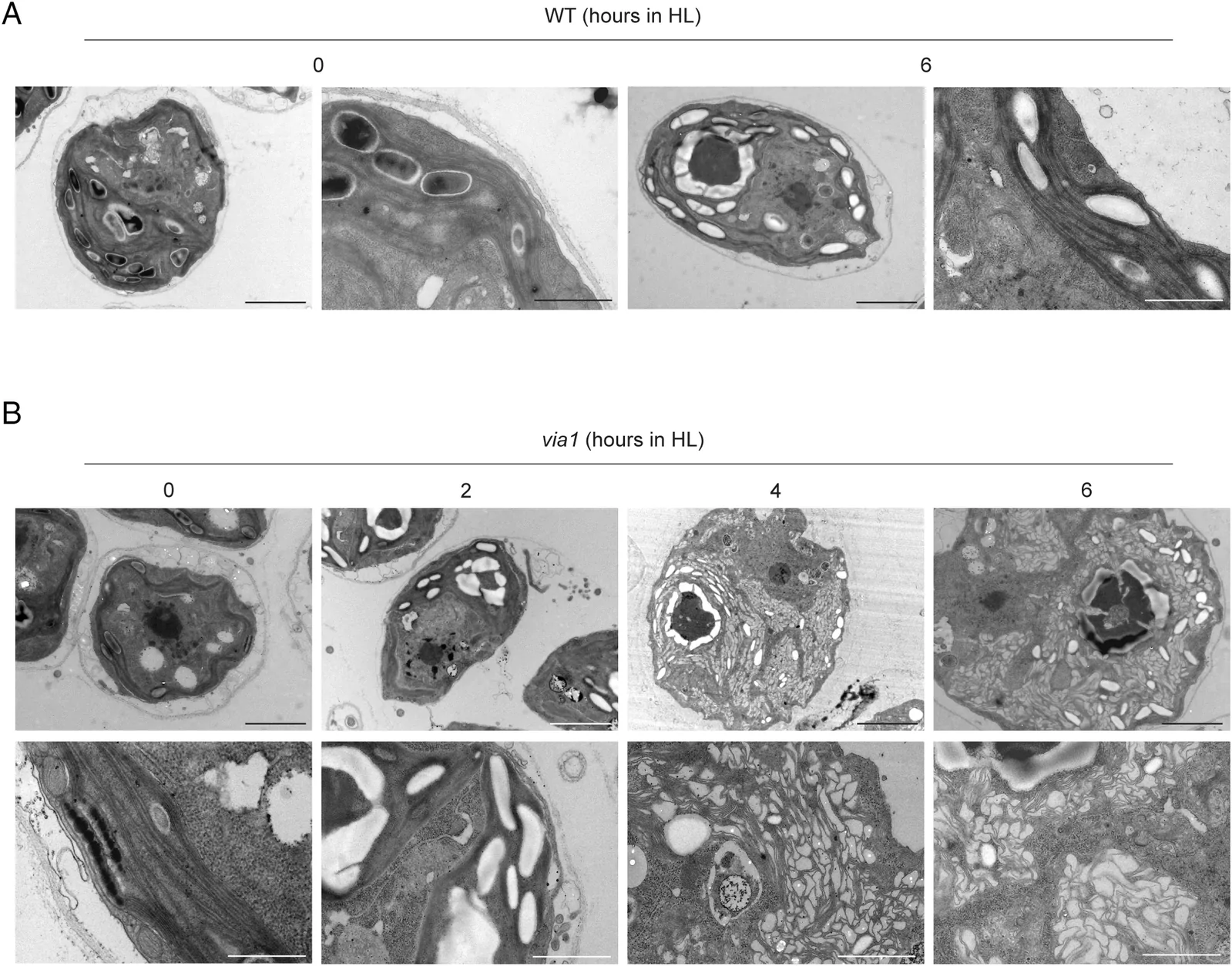
VIA1 is required for thylakoid membrane stability. (*A*) Representative TEM images of WT cells captured at 0 and 6 h of HL exposure (∼1,000 µmol photons m^−2^ s^−1^). Scale bar correspond to 2 µm for whole-cell images and 1 µm for zoomed-in images. (*B*) Representative TEM images of *via1*cells at 0-, 2-, 4-, and 6-h of HL exposure (∼1,000 µmol photons m^−2^ s^−1^). Scale bar correspond to 2 µm for the first row, 500 nm for the first image in the lower row, and 1 µm for the remaining images.

To determine the underlying cause for the HL sensitivity in *via* mutants, we examined chloroplast ultrastructure by transmission electron microscopy (TEM). Thylakoids in *via1* cells became severely swollen as early as 4 h after the HL onset (Fig. 3*B*), whereas they remained largely unaffected in WT until 6 h (Fig. 3*A* and *SI Appendix*, Fig. S4*C*). This phenotype resembled the HL-induced thylakoid defects reported for VIPP1 knockdown (14), albeit with delayed onset. To assess whether photosynthetic protein levels were also affected, we examined steady-state levels of core subunits of photosystem I, photosystem II, cytochrome b_6_f, and ATP synthase by immunoblotting at 2-hour intervals over an 8-hour HL time course. This analysis revealed comparable steady-state levels of core subunits of photosystem I, photosystem II, cytochrome *b_6_f*, and ATP synthase in WT and *via1* cells throughout the time course (*SI Appendix*, Fig. S4*D*). Thus, these results suggest that VIA1’s primary function is to maintain thylakoid membrane integrity under photooxidative stress, whereas the decline in photosynthetic activity observed at later time points is likely a secondary consequence.

### VIA1 Is Part of a Chloroplast Complex That Localizes to the Thylakoid Membranes and Contains VIPP1.

**Fig. 4.**
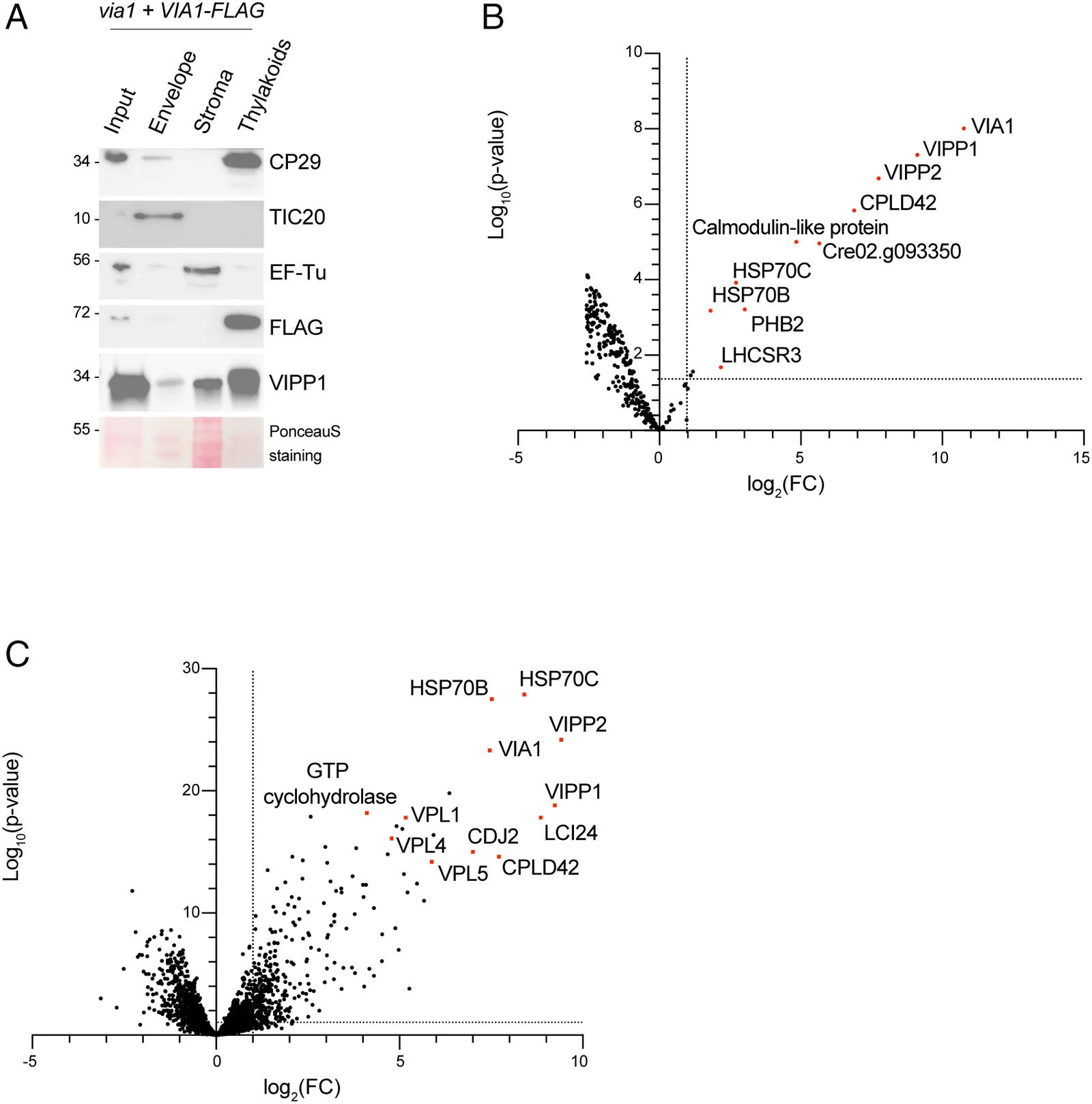
VIA1 interacts with VIPP1 and localizes to thylakoid membranes. (*A*) Chloroplast subfractionation on a two-step sucrose gradient followed by immunoblotting for VIA1–FLAG and VIPP1. CP29 (thylakoids), TIC20 (envelope), and Ef-Tu (stroma) were used as fraction markers to assess sample purity. (*B*) Volcano plot displaying log_2_ fold change versus –log_10_ *P* for each confidently quantified protein in FLAG coimmunoprecipitations from *via1* + VIA1-FLAG strain (three biological replicates) compared with controls (WT lysate incubated with FLAG–M2 beads). (*C*) Volcano plot of the VIPP1 coimmunoprecipitations from WT, displaying log_2_ fold change versus –log_10_ *P* for each confidently quantified protein. Significantly enriched proteins of interest are highlighted in red.

Given the membrane ultrastructure defects observed under HL, we next asked where VIA1 localizes within the chloroplast and which proteins associate with it. To enable biochemical analyses, we complemented *via1* mutant cells by nuclear transformation with *VIA1* genomic DNA (including endogenous regulatory regions) fused to a C-terminal 3 × FLAG tag (32, 33). We confirmed VIA1–FLAG expression by immunoblotting (*SI Appendix*, Fig. S5*A*), and complementation of the mutant phenotype was verified by HL growth assays (*SI Appendix*, Fig. S5 *B* and *C*). Chloroplast subfractionation showed that VIA1 is specifically enriched in the thylakoid fraction (Fig. 4*A*). We then performed FLAG immunoprecipitation coupled to mass spectrometry (IP–MS) under standard light and HL conditions, using untagged WT as a negative control. Under HL, VIA1–FLAG was recovered as the top hit, and VIPP1 emerged as the most prominent coenriched interactor (Fig. 4*B* and *SI Appendix*, Fig. S6*A*). Additional enriched proteins included cpUPR- and stress-associated factors, namely VIPP2 (an early and selective marker of cpUPR activation) and HSP70B (a chloroplast-localized chaperone), both previously reported as VIPP1 interactors (34, 35).

Considering the presence of VIPP1 in the VIA1 interactome and the similar phenotype of the *vipp1* knockdown (*vipp1-kd) a*nd the *via1* mutants, we hypothesized that VIA1 might form a complex with VIPP1 and modulate its membrane remodeling activity in stress conditions. To further test the formation of a VIA1:VIPP1 complex, we conducted the reciprocal immunoprecipitation using VIPP1 as the bait (Fig. 4*C* and *SI Appendix*, Fig. S6*B*). Cell lysates from a WT Chlamydomonas strain were incubated either with protein-A Dynabeads coupled with a polyclonal anti-VIPP1 antibody or with uncoupled Dynabeads (negative control). The immunoprecipitation was conducted in normal light and HL conditions in WT and *via1* backgrounds. A total of 377 proteins were identified as potential VIPP1 interactors upon HL stress across all three technical replicates, with VIPP2 and VIPP1 being the most abundant. Heat Shock Protein 70B (HSP70B) and chloroplast DNAJ-like chaperone 2 (CDJ2) were also abundantly present. In addition, several uncharacterized proteins were detected, including VPL1–6 and VPL10, which were previously identified in a VIPP1 proximity-labeling study (35). Importantly, peptides corresponding to VIA1 were significantly enriched in all samples, confirming the interaction between VIA1 and VIPP1. Together, these findings provide converging evidence that VIA1 and VIPP1 are binding partners in vivo.

### The Two Winged-Helix Domains in VIA1 Mediate the Interaction With VIPP1.

**Fig. 5.**
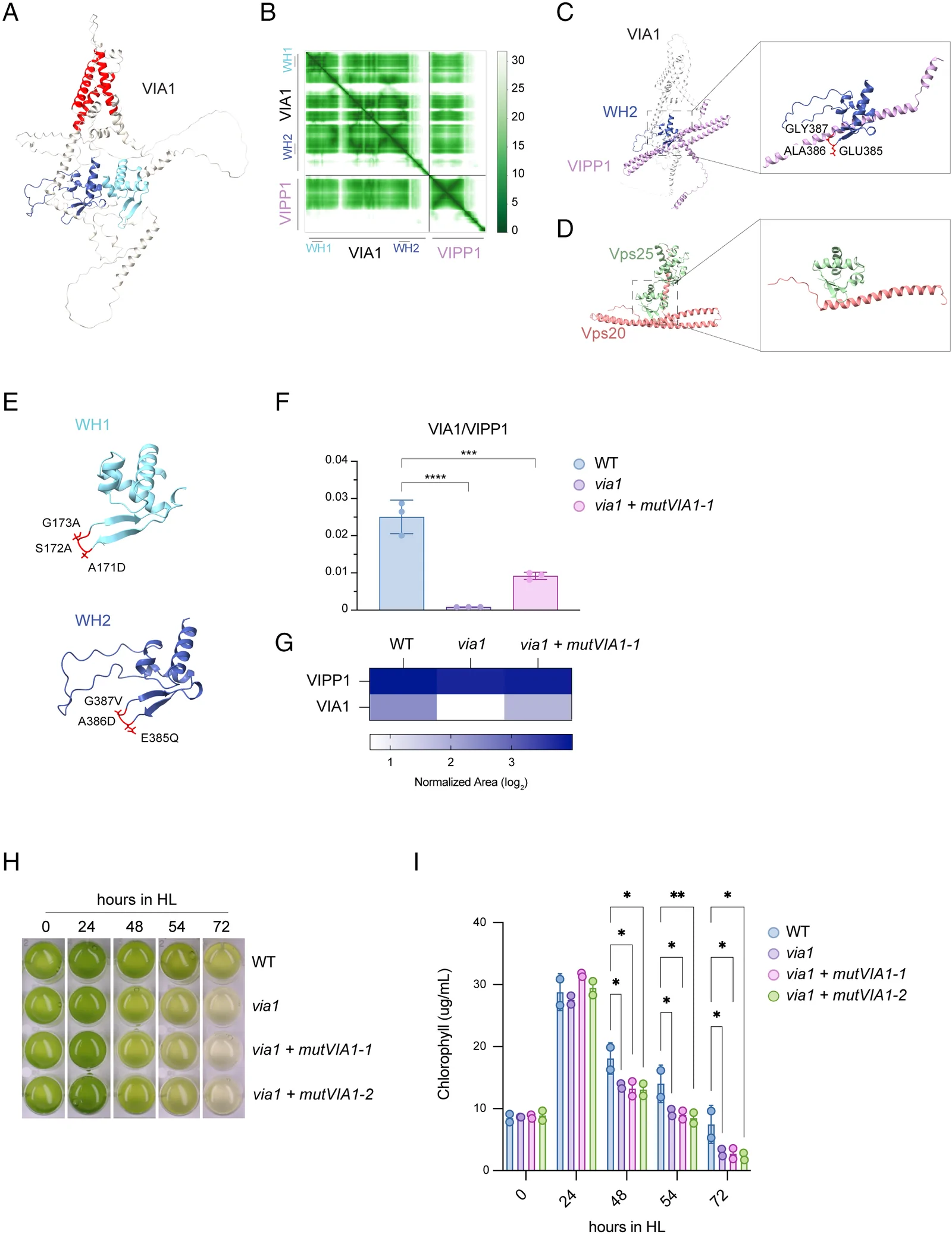
VIA1 winged-helix domains (WHs) mediate VIPP1 binding and VIA1 function. (*A*) AF3 structural prediction of VIA1. WH1 and WH2 are shown in cyan and blue, respectively; the three predicted transmembrane domains are highlighted in red. (*B*) Predicted Aligned Error plot (PAE) of the AF3 prediction in (*A*), with WH residues indicated. (*C*) AF3 prediction of the VIA1–VIPP1 complex, with a zoom-in on the predicted interaction interface involving VIA1-WH2 and the first α-helix of VIPP1. (*D*) Crystal structure of human Vps25 and Vps20 (ESCRT-II and ESCRT-III proteins, respectively) illustrating a similar interaction interface to that predicted for VIA1 and VIPP1 (PDB 3HTU). (*E*) AF3 prediction of the two WHs showing the loop residues targeted for mutagenesis in *mutVIA1*, with the corresponding amino acid substitutions indicated. (*F*) Histogram showing the VIA1/VIPP1 abundance ratio in VIPP1 coimmunoprecipitations from WT, *via1*, and *via1* + *mutVIA1* (three technical replicates). (*G*) Heatmap of log_2_ normalized areas for VIA1 and VIPP1 across the three strains used for VIPP1 coimmunoprecipitations in (*F*). (*H*) Representative images of liquid TAP cultures of WT, *via1*, *via1* + *mutVIA1-1*, and *via1* + *mutVIA1-2* at various time points during a 72-h HL treatment (∼800 µmol photons m^−2^ s^−1^). *via1* + *mutVIA1-1* and *via1* + *mutVIA1-2* are two independent transformants expressing the same mutated *VIA1* transgene. (*I*) Chlorophyll measurements from the assay in (*H*) (three biological replicates). The significance was assessed via Two-Way ANOVA and Tukey’s multiple comparisons analysis.

Having established that VIA1 associates with VIPP1 in vivo, we next sought to define the mechanistic basis of this interaction. VIA1 is predicted to contain three transmembrane helices (DeepTMHMM 1.0 (36); Fig. 5*A* and *SI Appendix*, Fig. S13*E*), consistent with its enrichment in thylakoid fractions and suggesting direct membrane anchoring. AlphaFold3 (AF3) (37) modeling additionally identified two winged-helix domains (WH1 and WH2), a fold comprising a three-helix bundle and a four-stranded β-sheet. Although winged-helix folds are classically linked to nucleic acid binding (38), they are also found in ESCRT-II subunits such as Vps25 (26), where they provide protein–protein interaction surfaces for ESCRT complex assembly and ESCRT-III engagement (39, 40). This raised the possibility that VIA1 WHs might form the binding interface for VIPP1.

Consistent with this hypothesis, AF3 prediction of the VIA1–VIPP1 complex placed the primary interface between VIA1-WH2 and the first α-helix of VIPP1 (ah1) (Fig. 5 *B* and *C*). Two orthogonal analyses supported this interface: Alphabridge (41) identified stabilizing interchain contacts enriched between VIA1-WH2 and VIPP1-ah1 (*SI Appendix*, Fig. S7*C*), and Pickluster (42) highlighted a recurrent interaction patch at the same region across models (*SI Appendix*, Fig. S7*D*). Notably, in the model, the WH2 loop between the first and second β-strands was predicted to wrap around VIPP1-ah1, resembling ESCRT-II/ESCRT-III binding geometries [for example, Vps25–Vps20 (39); Fig. 5*D*].

To test the AF3 model, we generated a VIA1 variant (mutVIA1) by site-directed mutagenesis, introducing three point mutations in the predicted VIPP1-interacting loop of each WH domain (*SI Appendix*, Fig. S7 *D* and *E*). Specifically, we introduced A171D/S172A/G173A in WH1, and E385Q/A386D/G387V in WH2 (Fig. 5*E*)—substitutions predicted by AF3 and Alphabridge analysis to disrupt the VIA1–VIPP1 interface (*SI Appendix*, Fig. S7 *B* and *C*). To minimize variability in expression levels arising from random nuclear genomic integration, we inserted the mutant *VIA1* transgene into a defined neutral landing site in the chloroplast genome of *via1* cells by biolistic chloroplast transformation (43, 44), and confirmed four independent transformants by RT–qPCR (*SI Appendix*, Fig. S7*F*).

To assess the impact of these mutations on the VIA1–VIPP1 interaction in vivo, we immunoprecipitated VIPP1 from WT, *via1*, and *via1* + *mutVIA1* cells. Mutant VIA1 expression was readily detected in input samples, but its coimmunoprecipitation with VIPP1 was strongly reduced compared with WT, indicating a substantially weakened association (Fig. 5 *F* and *G* and *SI Appendix*, Fig. S8 *A*–*B*). We next asked whether disrupting the VIA1–VIPP1 interaction compromises VIA1 function. In HL growth assays, the strains expressing the binding-defective VIA1 variant failed to rescue the *via1* mutant phenotype, exhibiting reduced chlorophyll accumulation relative to WT from ~48 h onward (Fig. 5 *H* and *I*). Together, these results demonstrate that the WH domains of VIA1 are required for robust association with VIPP1 in vivo, and that VIA1 function in HL acclimation depends on its ability to bind VIPP1.

### Structural Conservation of VIA1-WHs Underlies Cross-Species Functional Complementation.

**Fig. 6.**
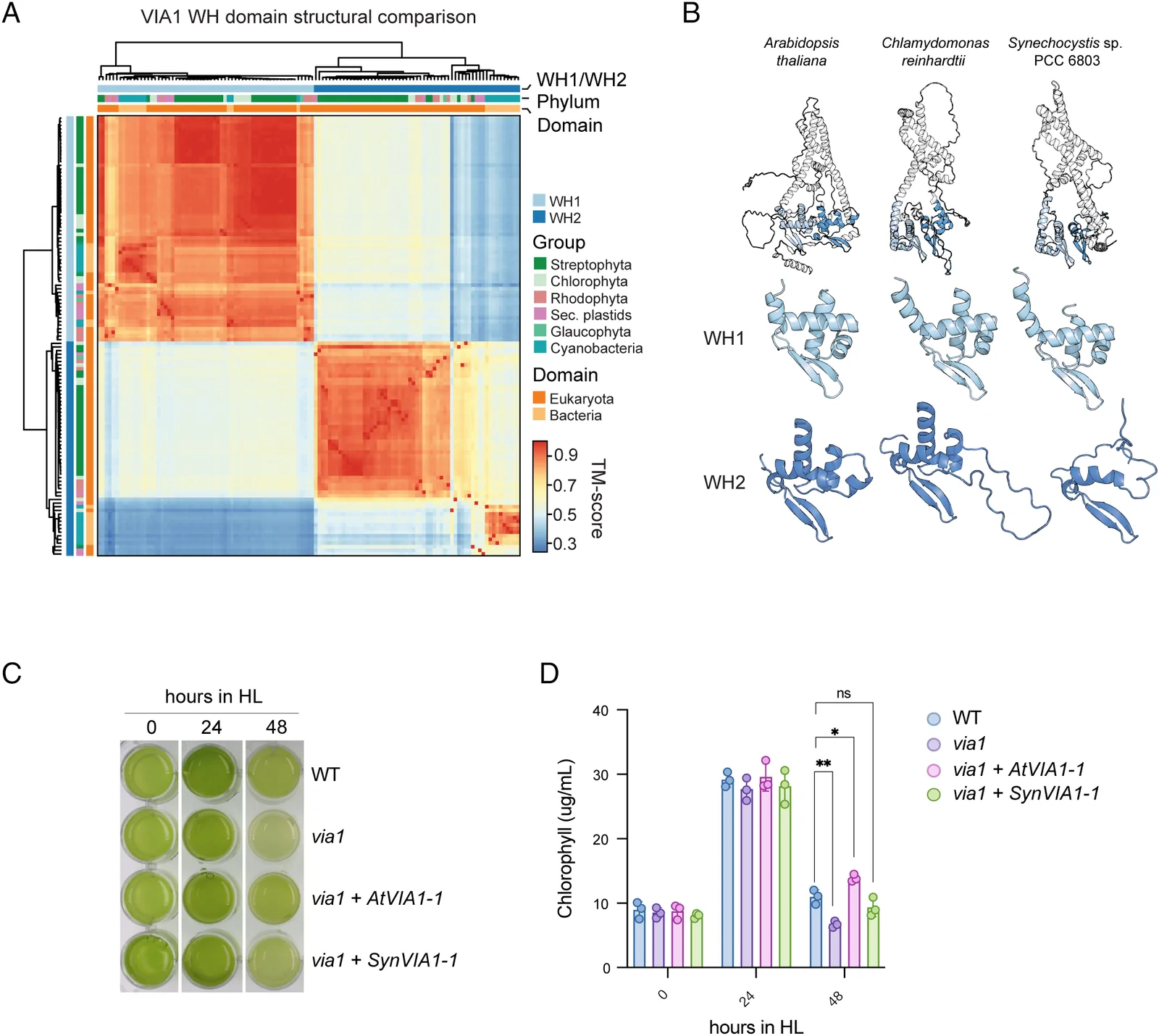
VIA1 structural conservation and cross-species complementation of the Chlamydomonas *via1.* (*A*) Structural comparison of WHs from representative eukaryotic and bacterial VIA1 proteins. Structures were predicted using AF2, and WHs were defined using Chlamydomonas WH1 as a reference. Domain position and taxonomic information are noted with colored lines. TM-score, template modeling score. (*B*) AF2-predicted full-length VIA1 structures and corresponding WHs from *A. thaliana* (UniProt ID: Q8GW20), *C. reinhardtii* (A0A2K3DKE5), and *Synechocystis sp.* PCC 6803 (P72990). (*C*) Representative images of liquid TAP cultures of WT, *via1*, *via1* + *AtVIA1*, and *via1* + *SynVIA1* at the indicated time points during a 48-h HL treatment (∼800 µmol photons m^−2^ s^−1^). (*D*) Total chlorophyll content measured over the HL time course shown in (*C*). Statistics were performed by two-way ANOVA with Tukey’s multiple-comparisons test; ns, not significant.

As shown in Fig. 1*B*, VIA1 is conserved across the green lineage and is also present in cyanobacteria and diatoms. To link this sequence conservation to structural and functional conservation, we analyzed VIA1 homologs using AlphaFold2 (AF2) structural predictions. In all species examined, VIA1 contains two WHs, one at the N terminus (WH1) and one at the C terminus (WH2) (Fig. 6*A*). WH1 is highly conserved across both eukaryotic and prokaryotic homologs, whereas WH2 is broadly conserved among eukaryotes but shows greater structural divergence in prokaryotes (Fig. 6 *A* and *B*). Despite this variation, the consistent presence of both WHs across lineages suggests that they are ancestral features of VIA1, likely predating plastic endosymbiosis and already present in the cyanobacterial ancestor.

Given the structural similarity between VIA1 from Chlamydomonas, Arabidopsis, and Synechocystis, we next asked whether orthologues from these distant lineages can functionally complement the Chlamydomonas *via1* mutant phenotype. To test this, untagged VIA1 orthologues from Arabidopsis (*AtVIA1*) and Synechocystis (*SynVIA1*) were integrated into the Chlamydomonas chloroplast genome of *via1* mutant cells by biolistic transformation and comparable transcript accumulation of the introduced orthologs was confirmed by RT–qPCR (*SI Appendix*, Fig. S9 *A* and *B*).

In HL growth assays, expression of both AtVIA1 and SynVIA1 rescued the *via1* photosensitivity phenotype, with chlorophyll levels returning to near-WT values after 48 h of HL exposure (Fig. 6 *C* and *D*). Notably, SynVIA1 rescue was weaker, consistent with its greater structural divergence in WH2.

These results suggest that VIA1 function is at least partially conserved across algae, land plants, and cyanobacteria. Nevertheless, because heterologous chloroplast expression may alter VIA1 protein abundance or its stoichiometry relative to endogenous VIPP1, differences in rescue efficiency should be interpreted with caution.

### VIA1 Interaction With VIPP1 and Its Role in Stress Protection are Conserved in *Synechocystis sp.* PCC 6803.

**Fig. 7.**
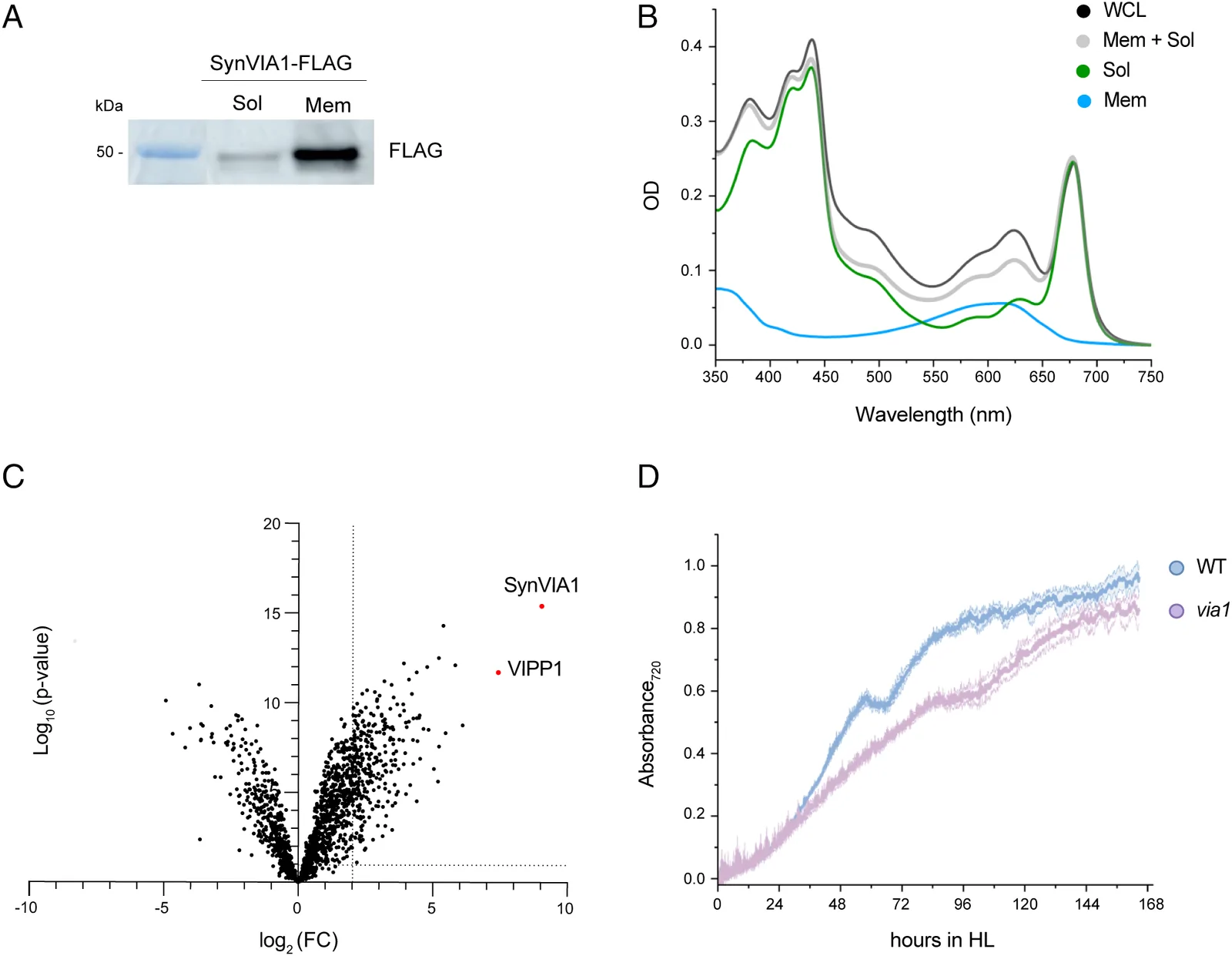
The VIA1–VIPP1 interaction and its role in stress response are conserved in *Synechocystis sp.* PCC 6803. (*A*) Whole-cell lysate (WCL) prepared from VIA1-FLAG cells was fractionated into soluble (Sol) and membrane (Mem) fractions by ultracentrifugation. A 1/10 aliquot of each fraction was subjected to FLAG immunoblot analysis. (*B*) Absorption spectra of the fractions described in (A). The WCL exhibits absorbance peaks in both the phycobilisome (PBS, associated with the soluble fraction) region (620 nm, gray dashed line) and the chlorophyll (Chl, membrane fraction) region (680 nm, gray dashed line). The soluble fraction shows a prominent peak in the PBS region with little to no signal in the Chl region, whereas the membrane fraction displays a strong Chl peak with minimal PBS signal. (*C*) Volcano plot of proteins identified by VIA1–FLAG coimmunoprecipitation mass spectrometry (IP–MS) from Synechocystis cells expressing VIA1-FLAG. Each dot represents a confidently quantified protein. The x-axis shows log_2_ fold change (FLAG IP vs. untagged WT control) and the y-axis shows −log_10_(p value). Significantly enriched proteins of interest are highlighted in red. (*D*) Growth analysis of *via1* mutant and WT cells under high light conditions (HL; 200 µmol photons m^–2^ s^–1^). Data represent the mean optical density at 720 nm (OD_720_) of four independent biological replicates, with shading indicating ± SE.

The strong structural conservation of VIA1 (Fig. 6 *A* and *B*) prompted us to examine whether the VIA1–VIPP1 complex represents an evolutionarily conserved feature of photosynthetic organisms. Although this complex has been recently shown to be present in *A. thaliana* (24), it remained unclear whether it is also present in cyanobacteria. To test this, we generated a *Synechocystis sp.* PCC 6803 strain expressing a FLAG-tagged version of the VIA1 ortholog (SynVIA1–FLAG) (*SI Appendix*, Fig. S10*A*), confirmed its membrane localization by fractionation (Fig. 7 *A* and *B* and *SI Appendix*, Fig. S10 *B* and *C*) and performed FLAG immunoprecipitation followed by mass spectrometry, using an untagged strain as a negative control. SynVIA1 was the most strongly enriched protein, and VIPP1 was the top coenriched interactor, supporting conservation of the VIA1–VIPP1 association in cyanobacteria (Fig. 7*C* and *SI Appendix*, Fig. S11).

To assess whether SynVIA1 contributes to light-stress acclimation in *Synechocystis *sp.* PCC 6803*, we generated an insertional *via1* mutant (*SI Appendix*, Fig. S12 *A* and *B*) and compared its growth with WT under normal light (NL; 80 µmol photons m^–2^ s^–1^) and high light (HL; 200 µmol photons m^–2^ s^–1^). Under NL conditions, the *via1* mutant grew similarly to WT, reaching comparable final cell densities (*SI Appendix*, Fig. S12*C*). Under HL, however, the *via1* mutant exhibited a consistent growth defect (Fig. 7*D*). Whole-cell absorption spectra of HL-grown cultures further highlighted differences between the two strains: Whereas WT and *via1* cells showed similar absorption in the chlorophyll and phycobilisomes regions (~675 nm and ~630 nm, respectively), *via1* cells displayed reduced absorption in the carotenoid region (460 to 520 nm), suggesting an impaired HL acclimation response (*SI Appendix*, Fig. S12*D*).

## Discussion

Thylakoid membrane integrity is fundamental to photosynthetic function yet can be compromised, especially under saturating light conditions, when protein and lipid damage must be continuously repaired. VIPP1, an ESCRT-III-like protein, is one of the most intensively studied and highly conserved factors implicated in thylakoid biogenesis and maintenance across both bacterial and eukaryotic photosynthetic lineages. Here, by leveraging the chloroplast unfolded protein response (cpUPR)—a signaling pathway triggered by proteotoxic and membrane stress—we identified VIA1 as a VIPP1-interacting protein. The biological relevance of this interaction is underscored by its independent discovery in *A. thaliana* by Yilmazer et al. (24), confirming that the VIA1–VIPP1 module is conserved across photosynthetic eukaryotes.

Structurally, VIA1 contains two conserved winged-helix domains and engages the α1 helix of VIPP1 through an interface that is functionally required. This interaction architecture is conceptually reminiscent of ESCRT-II/ESCRT-III contacts that coordinate membrane remodeling in eukaryotic cells (45). However, VIA1 differs fundamentally from canonical ESCRT-II in both organization and apparent function. Eukaryotic ESCRT-II is a soluble, heterotetrameric cytosolic complex that assembles as a Y-shaped scaffold and promotes ESCRT-III recruitment and polymer formation at membranes (40, 46). In contrast, VIA1 is a membrane-embedded protein. This imposes a distinct topological constraint: if both WH domains engage VIPP1 from the same side of the membrane, VIA1 would require an additional membrane-spanning segment not evident in the current AF3 model. As shown in *SI Appendix*, Fig. S13, a conserved hydrophobic stretch between the first transmembrane helix and the second WH domain is a plausible candidate for this role in VIA1, although the presence of charged residues makes it atypical of a canonical transmembrane helix. Resolving the membrane topology of VIA1 and its precise mode of engagement with VIPP1 will be an important goal for future structural and biochemical studies.

The WH domains mediating VIPP1 interaction are ancestral features of VIA1, present in both prokaryotic and eukaryotic homologs, suggesting that the VIA1–VIPP1 interaction module predates plastid endosymbiosis. Consistent with this, VIA1 orthologs from Arabidopsis and Synechocystis partially rescue the Chlamydomonas *via1* mutant phenotype, whereas disruption of VIA1 in Synechocystis compromises growth under light stress, mirroring the functional requirement observed in Chlamydomonas. These results are further supported by recent work showing that Chlamydomonas VIA1 can functionally complement the Arabidopsis *via1* mutant (24).

At the same time, the incomplete rescue observed in cross-species complementation experiments is difficult to interpret unambiguously and warrants caution. It may reflect technical limitations linked to heterologous VIA1 expression, including altered transcriptional regulation, protein abundance, or suboptimal stoichiometry relative to endogenous VIPP1. Alternatively, it may indicate that VIA1–VIPP1 interaction has been adapted to lineage-specific cellular contexts. In support of this latter possibility, VIA1 localization and mutant phenotypes differ between Chlamydomonas and Arabidopsis. In Chlamydomonas, VIA1 localizes to thylakoid membranes, and loss of function causes thylakoid swelling under high light, consistent with a role in stress-responsive membrane remodeling. In Arabidopsis, by contrast, VIA1 has been reported to associate with the inner chloroplast envelope and appears to be particularly important for thylakoid network formation during leaf development, with mutant plants accumulating vesicular structures that may reflect defects in membrane fusion (24).

Taken together, our findings suggest the existence of an evolutionarily ancient VIA1–VIPP1 interaction module that has been retained across the green lineage while raising the possibility that this module has undergone lineage-specific functional diversification during the evolution of photosynthetic organisms. Defining the mechanistic consequences of VIA1–VIPP1 interaction remains an important future goal. In particular, in vitro reconstitution of the VIA1–VIPP1 complex on liposomes of defined size and lipid composition should allow direct tests of whether VIA1 modulates VIPP1’s capacity to remodel membranes and whether any effects depend on lipid composition or membrane curvature. Such experiments would provide mechanistic insight into how VIA1 contributes to thylakoid membrane homeostasis.

## Materials and Methods

### Growth Conditions.

*C. reinhardtii* strains were maintained on solid Tris-Acetate-Phosphate (TAP) media containing 1.6% [w/v] agar (USP grade, Thermo Fisher Scientific) and Hutner’s trace elements (Chlamydomonas Resource Center) at 22 °C under low light conditions (~30 µmol photons m^−2^ s^−1^). CLiP mutant strains harboring the CIB1 insertion cassette encoding the *APHVIII* paromomycin resistance marker (CrRS432, CrRS433; see *SI Appendix*, Table S2 for details) were maintained on solid TAP media supplemented with 10 µg mL^−1^ paromomycin. Unless otherwise stated, strains were grown in liquid culture under standard light conditions with an intensity of 80 µmol photons m^–2^ s^–1^ and in the absence of any antibiotics. *Synechocystis sp.* PCC 6803 strains were cultured in BG11 medium supplemented with 6 µg/ml Ferric ammonium citrate, and 5 mM glucose, continuous white light (8 µmol photons m^−2^ sec^−1^) provided by T5 fluorescent lights at 26 °C, shaken at 120 rpm. Before harvesting cells for MS analysis, they were adapted to a light intensity of 20 µmol photons m^−2^ sec^−1^ for 5 d. A complete list of strains used in this research can be found in *SI Appendix*, Table S2.

### Isolation and Genotyping of *C. reinhardtii* VIA1 Insertion Strains.

Insertion of the mutagenic cassette at the VIA1 locus in *via1-1* (exon 10) and *via1-2* (intron 7) was verified by PCR using locus-flanking primer pairs (oRS39/oRS40 and oRS37/oRS38, respectively) that amplify both the WT and mutated loci, together with cassette-specific primers (oRS01 and oRS30) for selective amplification of the insertion. Genomic DNA quality was confirmed by mating type-specific PCR, and all amplicons were verified by agarose gel electrophoresis; primer sequences are listed in *SI Appendix*, Table S5. Further details are provided in the *SI* *Appendix*.

### RNA Extraction and RT-qPCR for Gene Expression Analysis.

Total RNA was extracted from WT, *via1-1*, and *via1-2* liquid cultures in exponential phase grown under standard light (80 µmol photons m^−2^ s^−1^) as described previously (23). For cDNA synthesis, 3 µg total RNA and oligo(dT)_18_ were used with the Maxima First Strand cDNA Synthesis Kit (Thermo Fisher Scientific) following the manufacturer’s instructions. cDNA was diluted 1:10 prior to quantitative PCR. qPCR was performed using SYBR Green chemistry (Bio-Rad) according to the manufacturer’s instructions. Primer sequences used to amplify *VIA1* are provided in *SI Appendix*, Table S5. No-template controls (master mix without cDNA) were included to assess contamination and nonspecific amplification. Ct values were analyzed following the “eleven golden rules,” as described previously (47). Transcript abundance was normalized to the reference gene *GBLP* (Cre06.g278222). Two biological replicates were analyzed per strain, each measured in two technical replicates.

### Phylogenetics and Species Distributions.

VIA1 homologs were identified by iterative searches combining Diamond BLASTP and a custom HMMER profile against diverse eukaryotic and prokaryotic proteomes, with hits curated phylogenetically using MAFFT alignments and IQ-Tree; the final phylogeny was inferred under the LG+C60+F+I+R8 substitution model and visualized using FigTree v1.4 and ITOL v6. Species distribution was further assessed by MiniProt-based genome searches in taxa lacking VIA1 gene models, with homologs included after phylogenetic curation. All protein sequences, alignments, phylogenies, and datasets are available at https://figshare.com/s/c411a207630c1d30d379. Full methodological details are provided in the *SI* *Appendix*.

### Structural Comparisons.

To assess structural conservation in the winged helix domains across VIA1 homologs, predicted protein structures were generated using AlphaFold2 and the MMseqs2 database (*n* = 62) (37, 48). The highest-ranking model (*n* = 5) for each protein was selected and split at its midpoint, generating N- and C-terminal halves. To extract the winged helix (WH) domains, the N-terminal WH from *C. reinhardtii* was isolated in PyMOL v2.5.0 (residues 35 to 98) and aligned to both the N- and C-terminal regions of each predicted VIA1 structure using TM-align v20220412 (query coverage > 50%) (49). Aligned regions were extracted, and pairwise TM-scores were calculated using TM-align. Predicted protein structures were visualized in PyMOL.

### High Light Assay and Time Course.

*C. reinhardtii* cultures were normalized to 8 µg chlorophyll mL^–1^ and exposed to high light (~800 µmol photons m^–2^ s^–1^) using a Plant LED Grow Light (Phlizon, Cob Series 3000W); tubes were randomly repositioned every 24 h to minimize positional effects, and chlorophyll content was monitored at indicated time points. For time-course immunoblot experiments, 5 mL aliquots were collected at 2 h intervals over 8 h of HL exposure, pelleted, and stored at –20 °C for subsequent protein extraction. Full methodological details, including culture preparation and light intensity calibration, are provided in the *SI* *Appendix*.

### TEM.

*C. reinhardtii* cells were subjected to high light (1,000 µmol photons m^–2^ s^–1^) for 0, 2, 4, and 6 h, and aliquots were fixed overnight in 2% paraformaldehyde/2% glutaraldehyde in 0.1 M sodium cacodylate buffer (pH 7.4). Samples were postfixed in 2% osmium tetroxide, dehydrated through a graded acetone series, and embedded in Agar 100 resin; 70 nm sections were poststained with uranyl acetate and Reynolds lead citrate. Micrographs were acquired on a FEI Morgagni 268D operated at 80 keV and equipped with a Mega View III CCD camera. Full methodological details are provided in the *SI* *Appendix*.

### Chloroplast Subfractionation.

Chloroplast subfractionation was performed using a protocol modified from (50), separating stroma, envelope, and thylakoid fractions by differential ultracentrifugation on a two-step sucrose gradient (1.3 M/1.8 M) prepared in 0.33 MS buffer. Cells were lysed by freeze–thaw cycling, and each fraction was collected based on its characteristic banding position and pelleted by ultracentrifugation. Fraction purity was assessed using compartment-specific marker proteins, and protein concentrations were determined by BCA assay prior to SDS–PAGE and immunoblot analysis. Full methodological details are provided in the *SI* *Appendix*.

### Membrane Subfractionation.

FLAG-VIA1 *Synechocystis sp.* PCC 6803 cells were lysed by French press, and cellular debris was removed by low-speed centrifugation to yield a whole-cell lysate fraction. Sequential ultracentrifugation steps (208,000×*g*) were used to separate soluble and membrane fractions, with a high-salt wash (150 mM NaCl) applied to the membrane pellet to remove peripherally associated proteins. Full methodological details are provided in the *SI* *Appendix*.

### Coimmunoprecipitation.

Cells were lysed by freeze–thaw cycling and membrane complexes solubilized with 1% digitonin prior to immunoprecipitation using either Protein A Dynabeads coupled to an anti-VIPP1 antibody or Magnetic Anti-FLAG M2 beads (Sigma-Aldrich), with non-antibody-coupled beads serving as negative controls. Anti-VIPP1 eluates were collected under denaturing conditions (70 °C, SDS buffer) for mass spectrometry, while FLAG eluates were collected under native conditions using 3 × FLAG peptide competition and pooled across three sequential elutions. Eluates for MS analysis were separated by SDS–PAGE, stained with colloidal Coomassie, and gel lanes were excised for in-gel digestion. Full methodological details are provided in the *SI* *Appendix*.

### Processing of Mass Spectrometry Samples, Analysis, and Data Processing.

Coomassie-stained gel bands were excised, destained, and subjected to in-gel reduction with DTT and alkylation with iodoacetamide prior to overnight trypsin digestion at 37 °C. Tryptic peptides were extracted by sequential formic acid addition and sonication, and all supernatants were pooled for LC–MS/MS analysis. Additional details, including NanoLC-MS/MS analysis and data processing, are provided in the *SI* *Appendix*.

### VIA1 and VIPP1 Structure Modeling.

CrVIA1 signal peptide sequence was predicted using TargetP 2.0 (28) and Wolf PSORT (29), while its transmembrane domains were predicted using DeepTMHMM (36). The predicted tertiary protein structure of VIA1 and VIPP1, as well as the interactions between the two proteins, was modeled by AF3 (51) using the protein sequences lacking their predicted chloroplast signal peptide. Graphical modifications on the protein structures were done using ChimeraX-1.4 (52).

AF3 structural models of VIA1–VIPP1 were used as input for Pickluster (42) and Alphabridge (41) analyses. Pickluster was employed to identify recurrent interaction patches across the top-ranked AF3 models, using default parameters, and visualized with ChimeraX-1.4. Alphabridge was used to detect interresidue contacts stabilized by hydrogen bonds and salt bridges between the two proteins, with default thresholds for contact distance and bond geometry. Interchain contacts identified by Alphabridge were visualized as chord diagrams representing connections between the corresponding interface regions.

Detailed descriptions of the methods for total protein extraction, immunoblot analysis, molecular cloning, chlorophyll fluorescence measurements, growth assay, backcross and tetrad dissection, and genetic transformation are provided in the *SI Appendix*.

## Supplementary Material

Appendix 01 (PDF)

## Data Availability

Protein sequences, alignments, phylogenies, and datasets used for phylogenetic analyses have been deposited to Figshare at https://figshare.com/s/c411a207630c1d30d379. The full phylogeny with species distribution is available via the Interactive Tree of Life (iTOL) at https://itol.embl.de/shared/1WjO4fmjYiy3z. The mass spectrometry proteomics data have been deposited to the ProteomeXchange Consortium via the PRIDE partner repository (53) with the dataset identifier PXD072529.
