# Supplementary material for: VIA1 is a conserved regulator of thylakoid membrane integrity that acts through VIPP1
Source: Proc Natl Acad Sci U S A. 2026 Aug 17;123(34):e2605816123. doi: 10.1073/pnas.2605816123 (PMC13505992; doi:10.1073/pnas.2605816123)
Supplement: Supplementary file 1 [file pnas.2605816123.sapp.pdf]

PNAS

Supporting Information for

VIA1 is a conserved regulator of thylakoid membrane integrity that acts through VIPP1

Pamela Vetrano[a,b]*, Kelsey Krall[c], Laura Martinez[a], Eleonora Traverso[d], Tomas Morosinotto[d], Nicholas A. T. Irwin[a], Yuval Mazor[c], and Silvia Ramundo[a,1]

[a] Gregor Mendel Institute, Austrian Academy of Sciences, Vienna BioCenter, Vienna 1030, Austria
[b] Vienna BioCenter PhD Program, Doctoral School of the University of Vienna and Medical University of Vienna, Vienna 1030, Austria
[c] Biodesign Institute, School of Molecular Sciences, Arizona State University, Tempe 85281, Arizona, USA
[d] Dipartimento di Biologia, Università di Padova, Padova 35131, Italy

[1] To whom correspondence may be addressed. Email: silvia.ramundo@gmi.oeaw.ac.at

This PDF file includes:

Figures S1 to S13
Tables S1 to S5
Material and methods

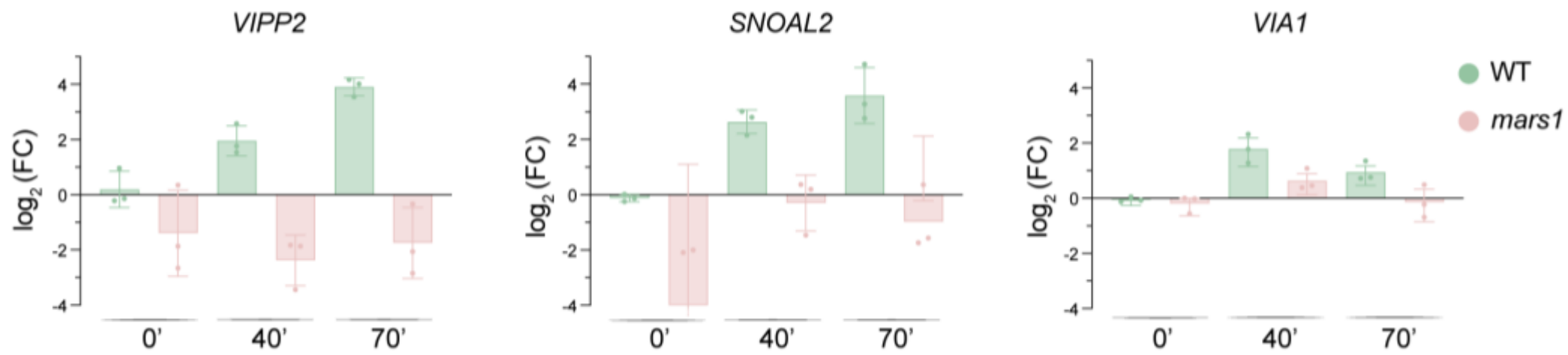

Figure S1. ***VIA1* expression is upregulated upon cpUPR activation in a MARS1-dependent manner.** WT and *mars1* knockdown cells were exposed to high light (>1000 µE) for 0, 40, and 70 minutes. The transcript levels of *VIA1* and two cpUPR marker genes (*VIPP2* and *SNOAL*) were assessed via RT-qPCR. Expression levels are shown as $\log_2$ fold change relative to WT.

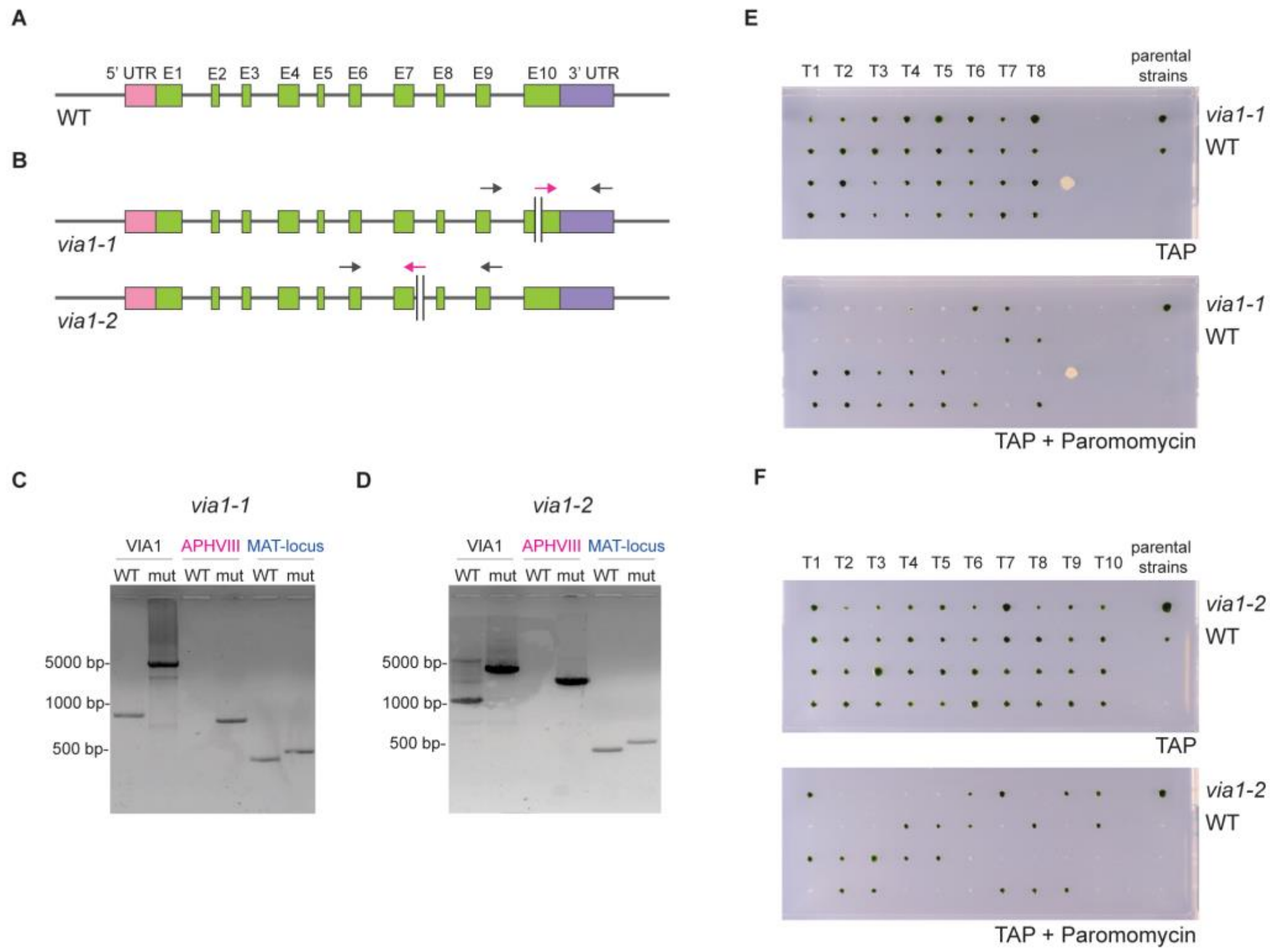

Figure S2. Validation of mutagenic insertion in Chlamydomonas *via1-1* and *via1-2* by PCR and segregation analysis. (A) Schematic representation of the *VIA1* transcript: exons are shown in green, 5'UTR in pink, and 3'UTR in purple. **(B)** Diagram illustrating the mutagenic insertion carrying the gene conferring paromomycin resistance (*APHVIII*) in *via-1* and *via1-2*. The mutagenic cassette (represented by black vertical lines) is located in exon 10 and intron 7, respectively. Its orientation is 5'→3' in *via1-1* and 3'→5' in *via1-2*. Arrows indicate primers used for the PCRs shown in panels C and D. **(C-D)** PCRs validating the mutagenic insertion in *via1-1* (panel C) and *via1-2* (panel D): The first PCR (*VIA1*) amplifies a region between black arrowheads in the diagrams. The size difference between WT and mutant amplicons reflects the presence of the cassette in mutants. The second PCR (*APHVIII*) amplifies the region between black and pink arrowheads, producing an amplicon only in mutants. The third PCR (*MAT-locus*) is a mating-type-specific PCR used as a control to ensure the quality of the employed genomic DNA. **(E-F)** Segregation analysis of the mutagenic cassette in tetrads (T1–T10) derived from backcrosses of *via1-1* **(E)** or *via1-2* **(F)** to the WT strain. Tetrads were re-arrayed on rectangular TAP agar plates. The upper section of each panel shows the tetrads growing on TAP agar, while the lower section displays the tetrads growing on TAP agar supplemented with paromomycin

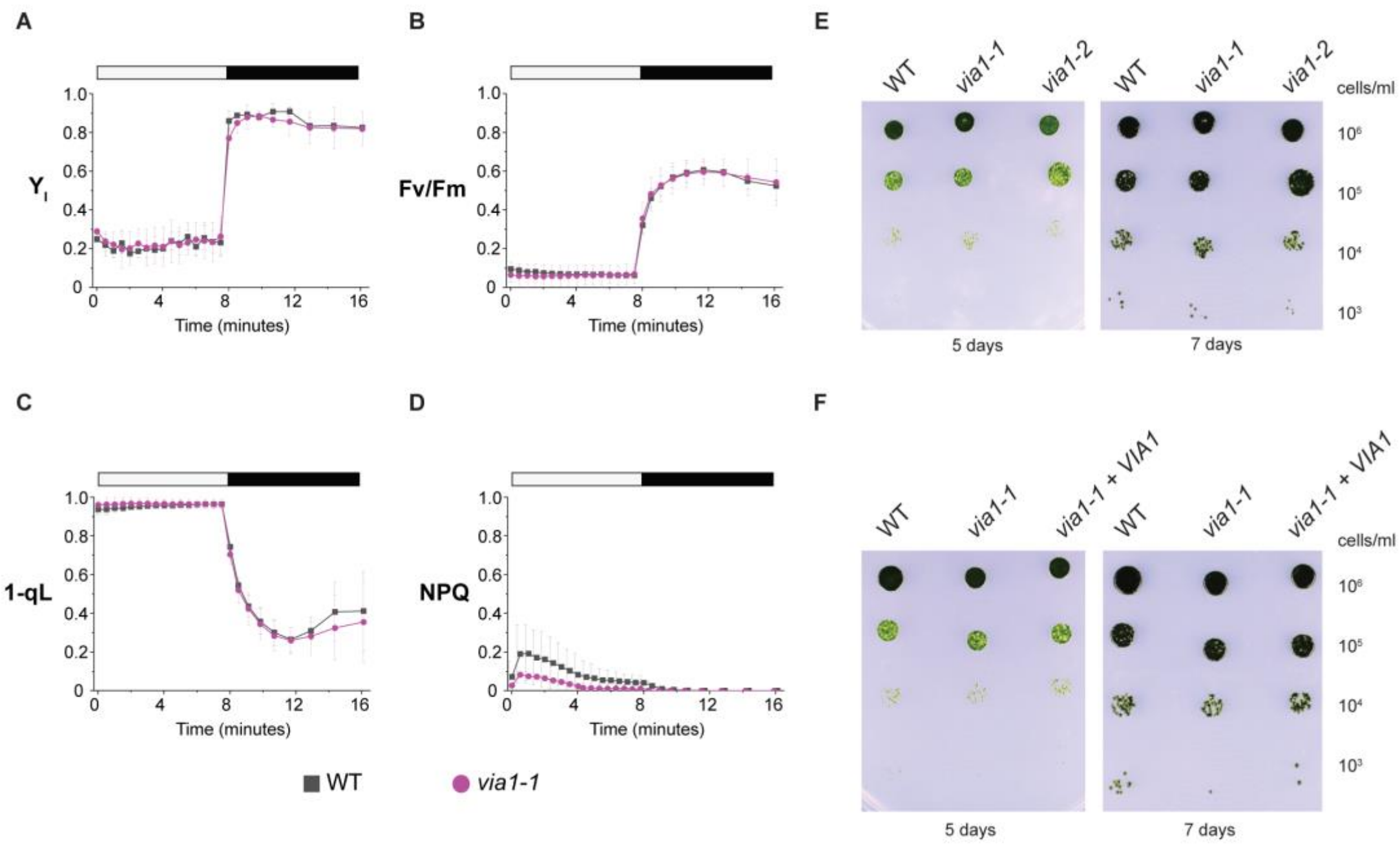

Figure S3. Photosynthetic measurements in Chlamydomonas WT and *via1-1* cells and growth assay in normal light conditions. (A-B) Photosystem I quantum yield (YI) **(A)** and Photosystem II quantum yield (Fv/Fm) **(B)** were monitored during an 8-minute exposure to actinic light at an intensity of 1000 µmol photons m^{-2} s^{-1}, followed by an 8-minute dark period. **(C-D)** The plastoquinone redox state was assessed by analyzing the fluorescence parameters 1-qL **(C)** and NPQ **(D)**. Data represent means ± standard deviation from four independent measurements. **(E-F)** Growth assay on TAP agar showing the growth of WT, *via1-1*, and *via1-2* **(E)**, and WT, *via1-1*, and *via1-1* + VIA1 **(F)** over 7 days. Liquid cultures were serially diluted to the indicated concentrations, spotted on TAP agar, grown under normal light (~80 µmol photons m^{-2} s^{-1}), and imaged 5- and 7-days post-spotting.

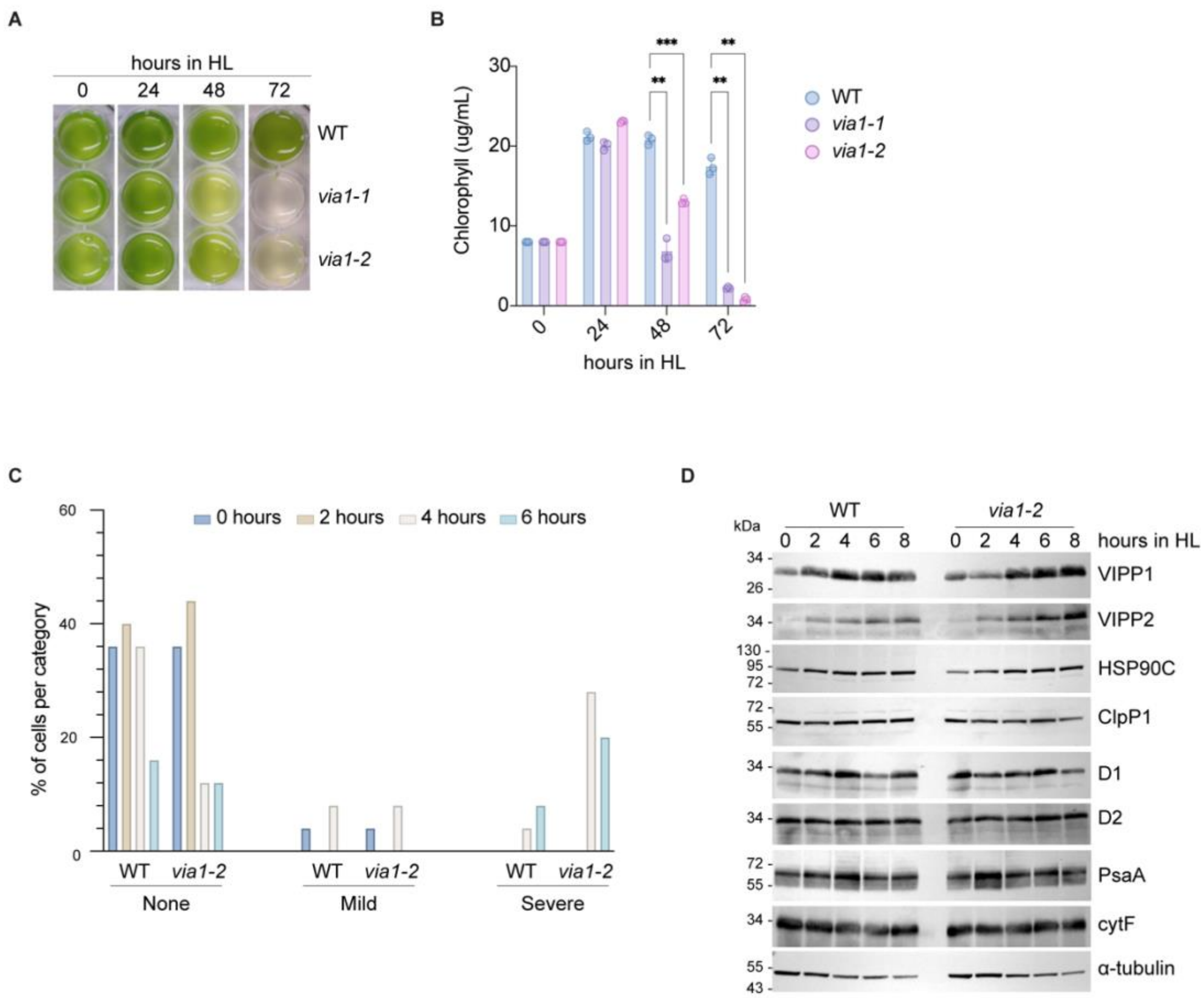

Figure S4. Additional characterization of *via1* mutant strains. (A) Images of liquid TAP cultures and **(B)** relative chlorophyll quantification of WT, *via1-1*, and *via1-2* at various time points during a 3-day HL treatment (~800 µmol photons m^{-2} s^{-1}). Three biological replicates were used. **(C)** Distribution of thylakoid swelling severity (none, mild, severe) in WT and *via1-2* cells imaged in Fig. 3A–B at the indicated time points during HL treatment. **(D)** Time-course immunoblot comparing the accumulation of VIPP1, D1, D2, PsaA, cytF, and ClpP1 in WT versus *via1-2* during HL treatment (0–8 h); α-tubulin serves as a loading control.

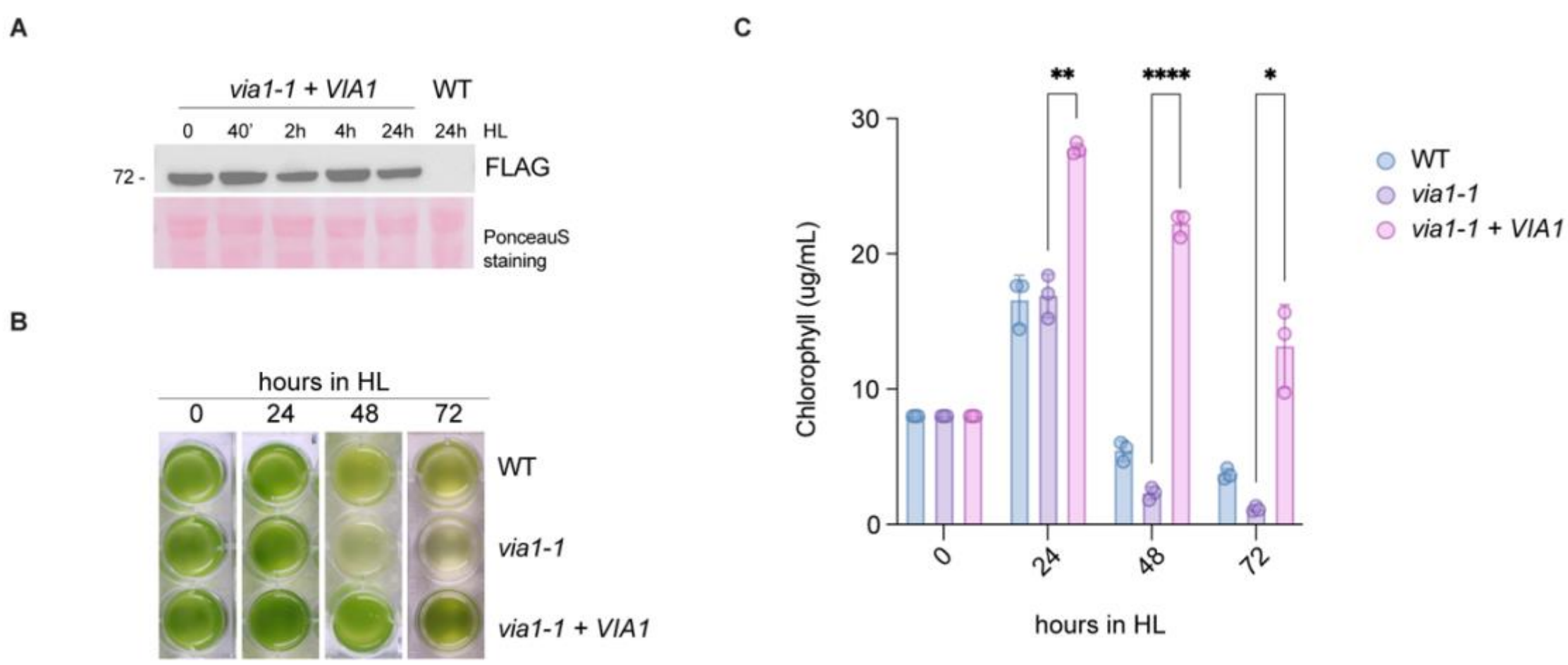

Figure S5. Phenotypic rescue of *via1* upon introduction of VIA1-FLAG transgene. (A) FLAG immunoblot analysis of total protein lysates from *via1-1 + VIA1-FLAG* cells subjected to HL treatment. **(B)** Representative images of liquid TAP cultures of WT, *via1-1*, and *via1-1 + VIA1-FLAG,* and **(C)** relative chlorophyll content measured at the indicated time points over a 3-day HL treatment (~800 µmol photons m^{-2} s^{-1}). Data are from three biological replicates.

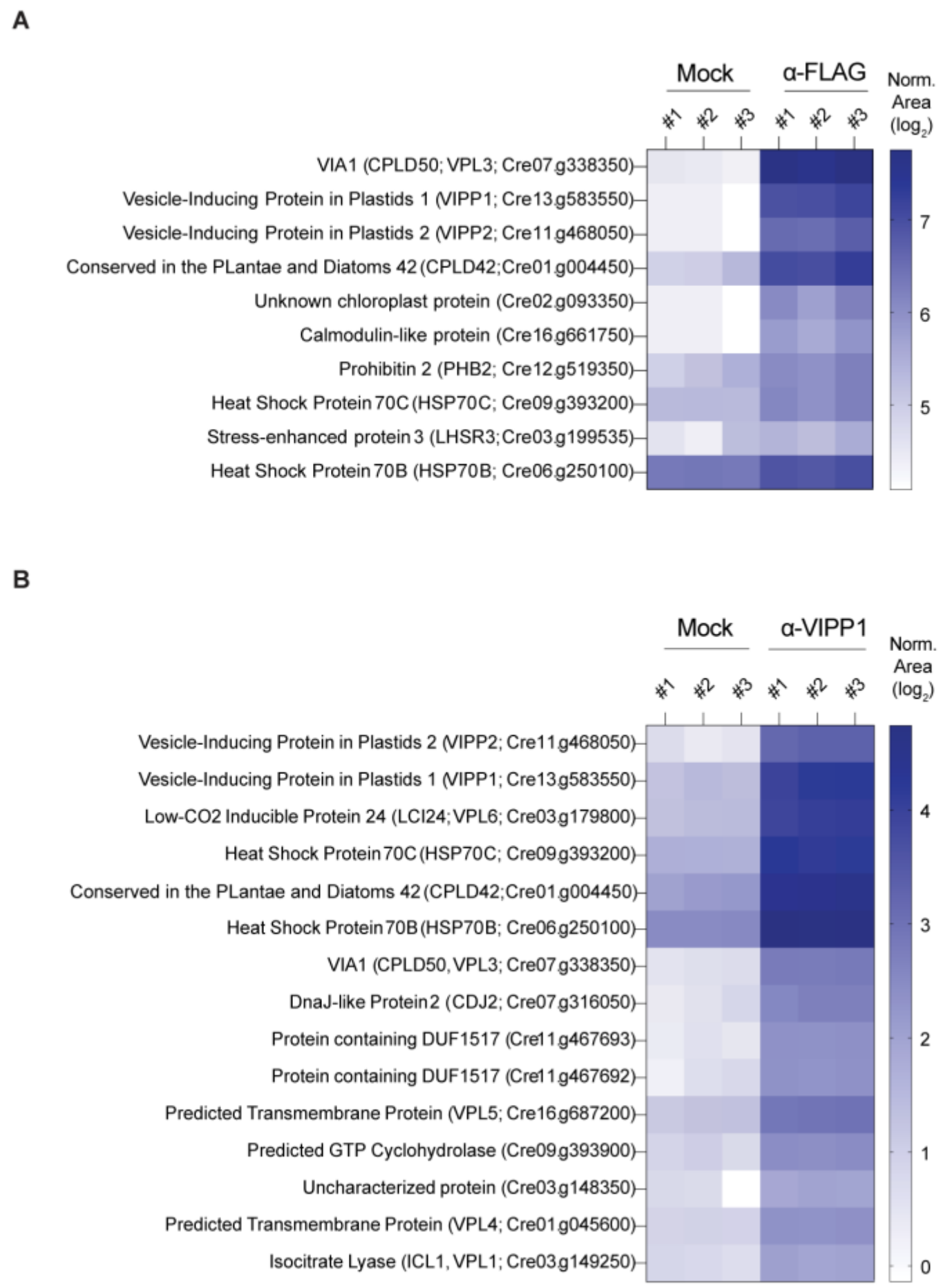

Figure S6. VIA1 interacts with VIPP1 in *Chlamydomonas reinhardtii*. (A) Heatmap showcasing proteins reproducibly enriched in FLAG co-immunoprecipitations from *via1* + *VIA1-FLAG* strain (three biological replicates) relative to control samples consisting of WT lysate incubated with FLAG–M2 beads. Heatmap colors indicate the $\log_2$ normalized area (relative abundance), with darker shading denoting higher relative abundance. **(B)** Heatmap of proteins significantly enriched in VIPP1 co-immunoprecipitations from WT cell, shown as $\log_2$ normalized area (relative abundance). Control samples consisted of WT lysates incubated with uncoupled Protein A Dynabeads.

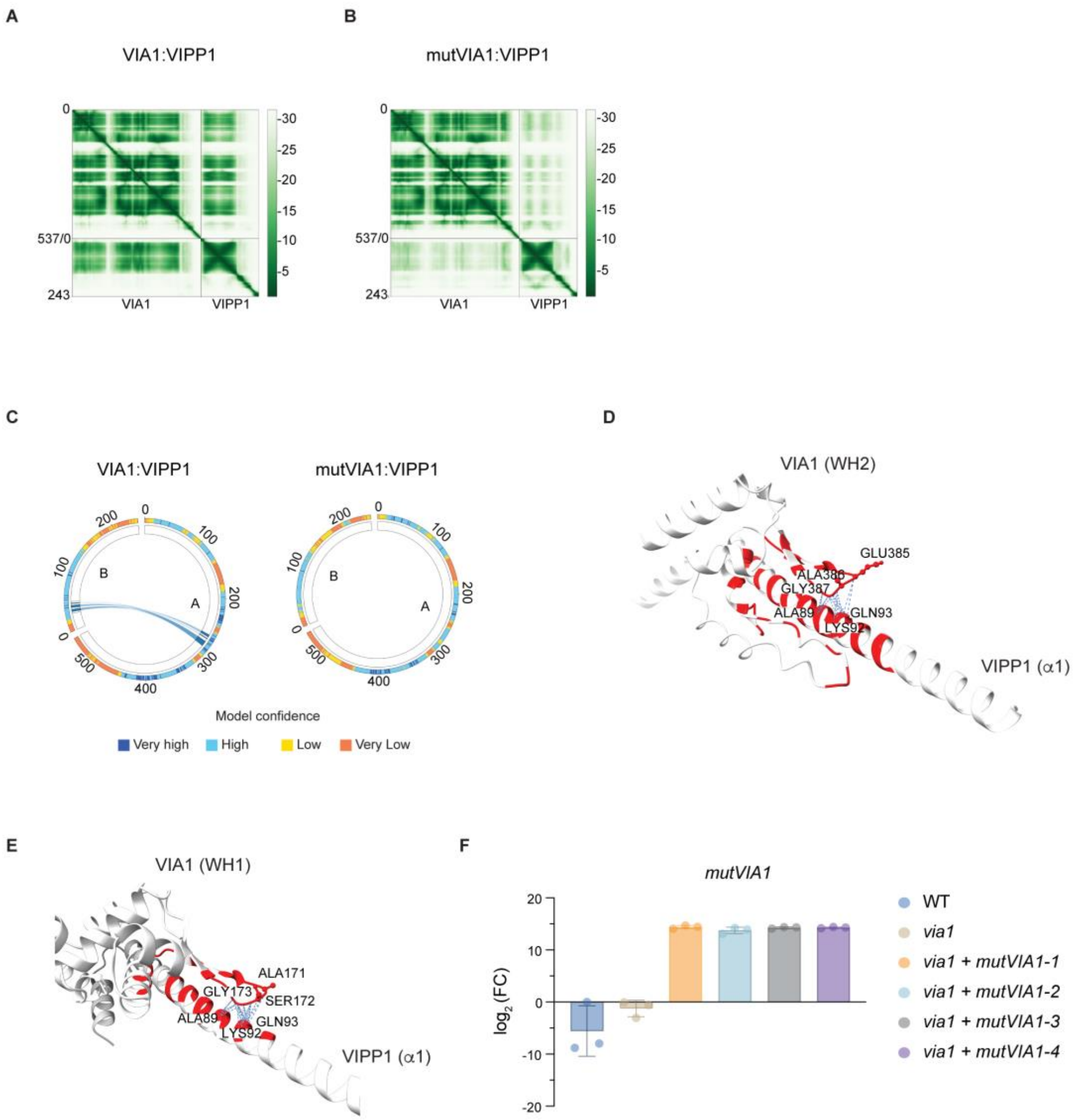

Figure S7. Structural prediction-guided design of VIA1 point mutants targeting the VIA1:VIPP1 interface. **(A-B)** Predicted Aligned Error (PAE) plots from the AlphaFold 3 models of the VIA1–VIPP1 **(A)** and mutVIA1–VIPP1 **(B)** complexes. **(C)** AlphaBridge chord diagrams of predicted residue–residue contacts between VIA1 and VIPP1 and between mutVIA1 and VIPP1. **(D-E)** PickLuster analysis of predicted interaction interfaces between VIPP1 and the WH2 **(D)** or WH1 **(E)** domain of VIA1, with predicted contacts mapped to loop regions targeted for point mutagenesis. **(F)** RT-qPCR analysis of *mutVIA1* transcript levels in WT, *via1*, and four independent *via1* + *mutVIA1* transformant strains. Expression levels are shown as $\log_2$ fold change relative to WT.

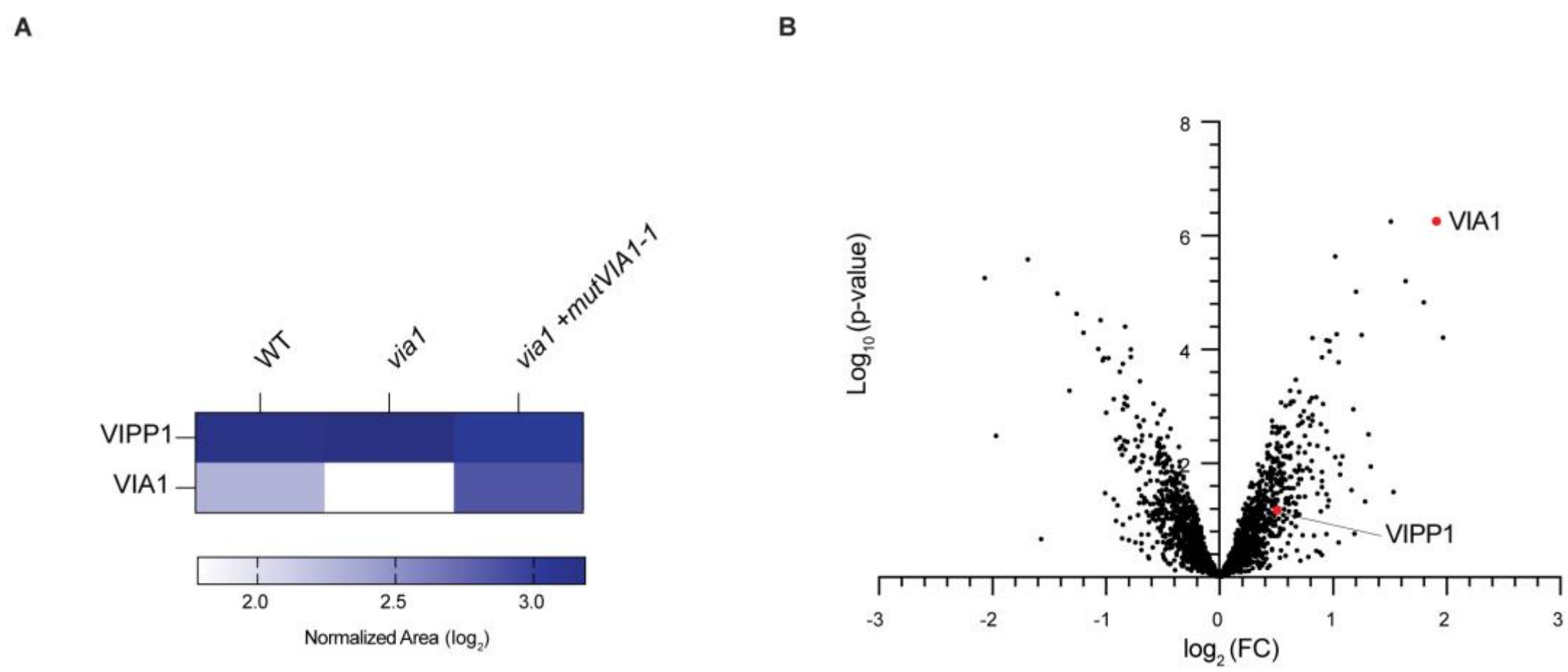

Figure S8. Comparative analysis of VIA1 and VIPP1 levels in VIPP1-co-immunoprecipitation inputs and eluates from WT, *via1*, and *via1* + *mutVIA1* strains. (A) Heatmap showing $\log_2$ normalized area (relative abundance) for VIA1 and VIPP1 in the VIPP1 co-immunoprecipitation inputs from WT, *via1*, and *via1* + *mutVIA1* strains. **(B)** Volcano plot showing the WT and *via1* + *mutVIA1* VIPP1 immunoprecipitation datasets. The x-axis shows $\log_2$ fold change for the WT/*via1* + *mutVIA1* ratio, and the y-axis shows $-\log_{10}$(p-value). VIA1 and VIPP1 are highlighted in red.

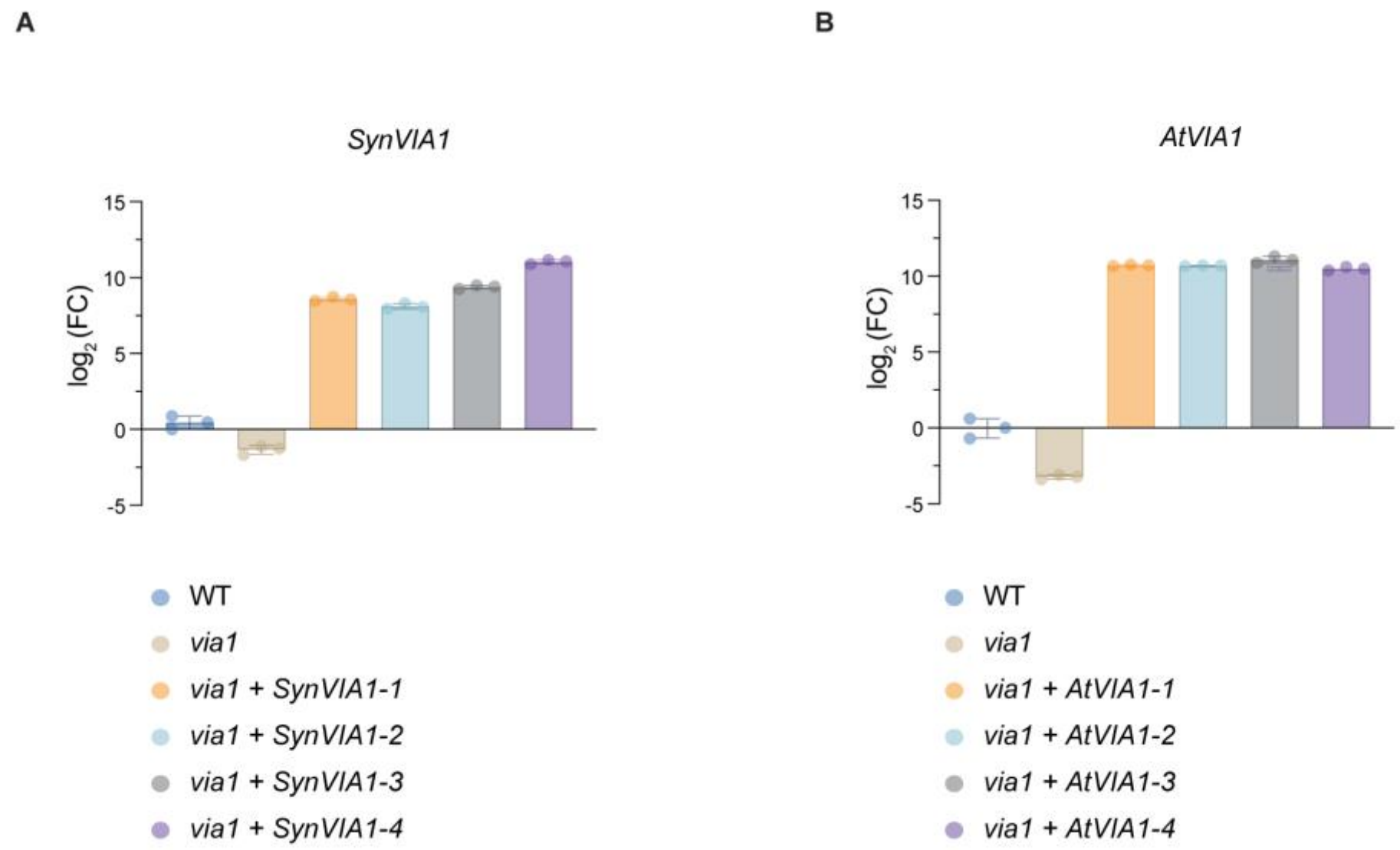

Figure S9. Expression of *VIA1* orthologs in cross-species complementation strains. qRT–PCR analysis of VIA1 ortholog expression from *Synechocystis sp.* PCC 6803 **(A)** and *Arabidopsis thaliana* **(B)** introduced into the Chlamydomonas *via1* mutant via biolistic chloroplast transformation. Transcript levels are shown as log_2 fold change relative to WT. Four independent transformants were analyzed for each ortholog.

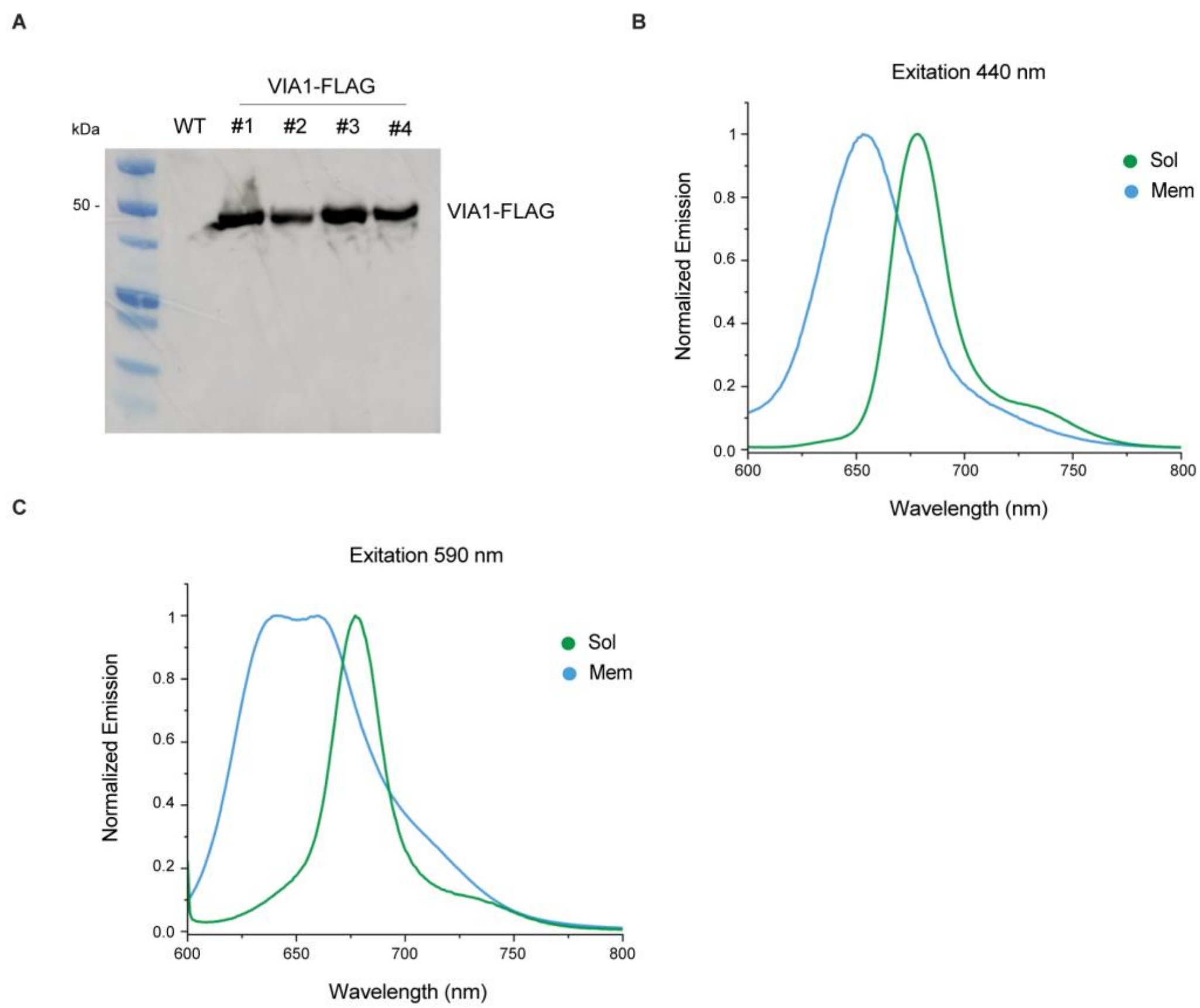

Figure S10. Generation and biochemical characterization of a VIA1-FLAG-tagged strain in *Synechocystis sp.* PCC 6803. (A) The endogenous *VIA* gene (*slr1603*) was tagged with a C-terminal 3XFlag. Four independent clones obtained after complete segregation of the tagged allele were verified by FLAG immunoblot analysis. Clone #1 was used in the subsequent experiments. **(B)** Normalized fluorescence emission spectra excited at 440 nm (Chl excitation) reveal trace Chl levels in the soluble fraction and confirm the absence of PBS signal in the membrane fraction. **(C)** Normalized fluorescence emission spectra excited at 590 nm (PBS excitation) confirm the absence of PBS in the membrane fraction and reveal only trace Chl levels in the soluble fraction.

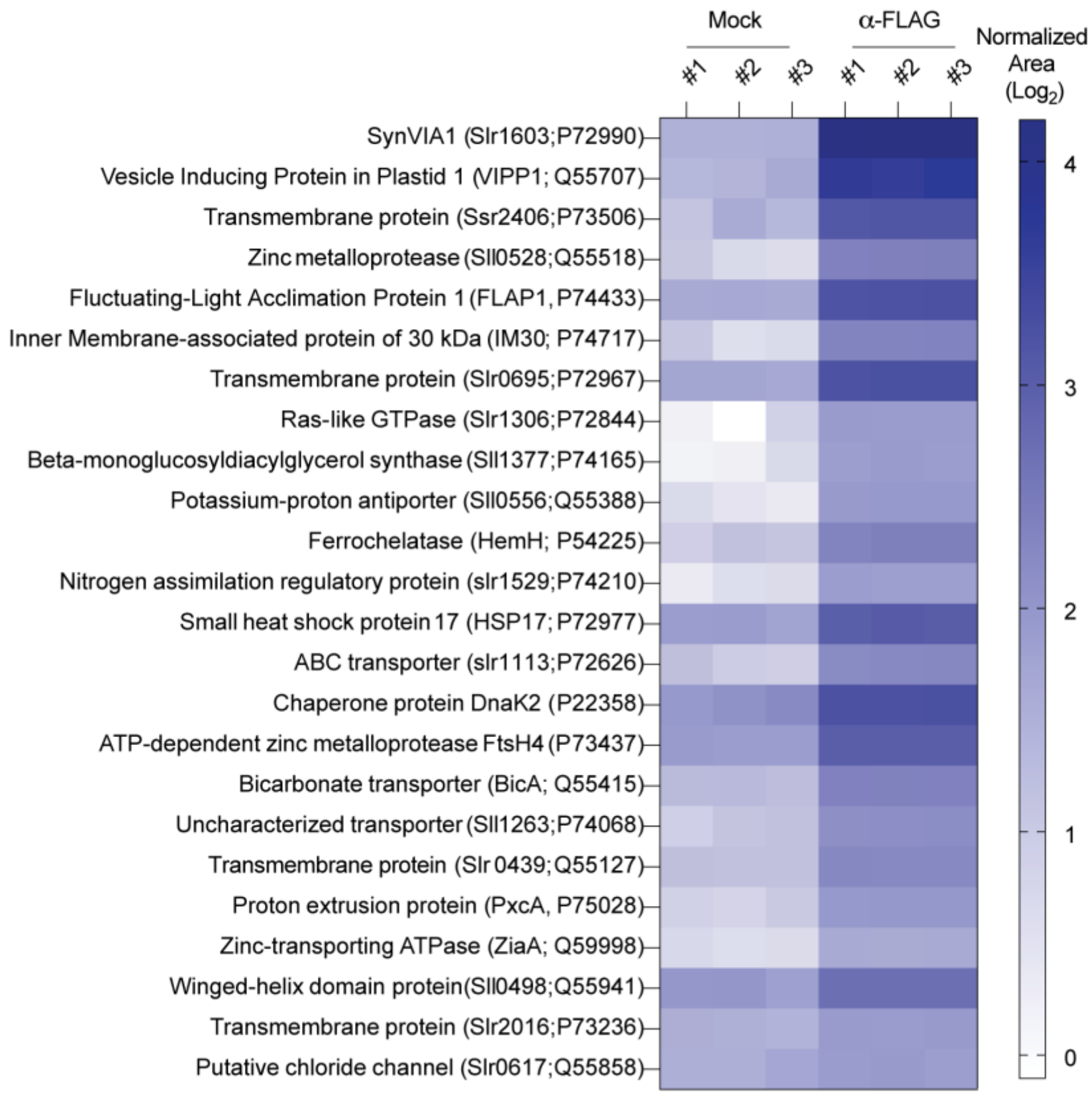

Figure S11. VIA1 interacts with VIPP1 in *Synechocystis sp.* PCC 6803. Heatmap of proteins reproducibly enriched in FLAG co-immunoprecipitations from a *Synechocystis* sp. PCC 6803 strain expressing SynVIA1-FLAG, relative to untagged WT control samples. Heatmap colors represent log_2 normalized area (relative protein abundance), with darker shading indicating higher abundance.

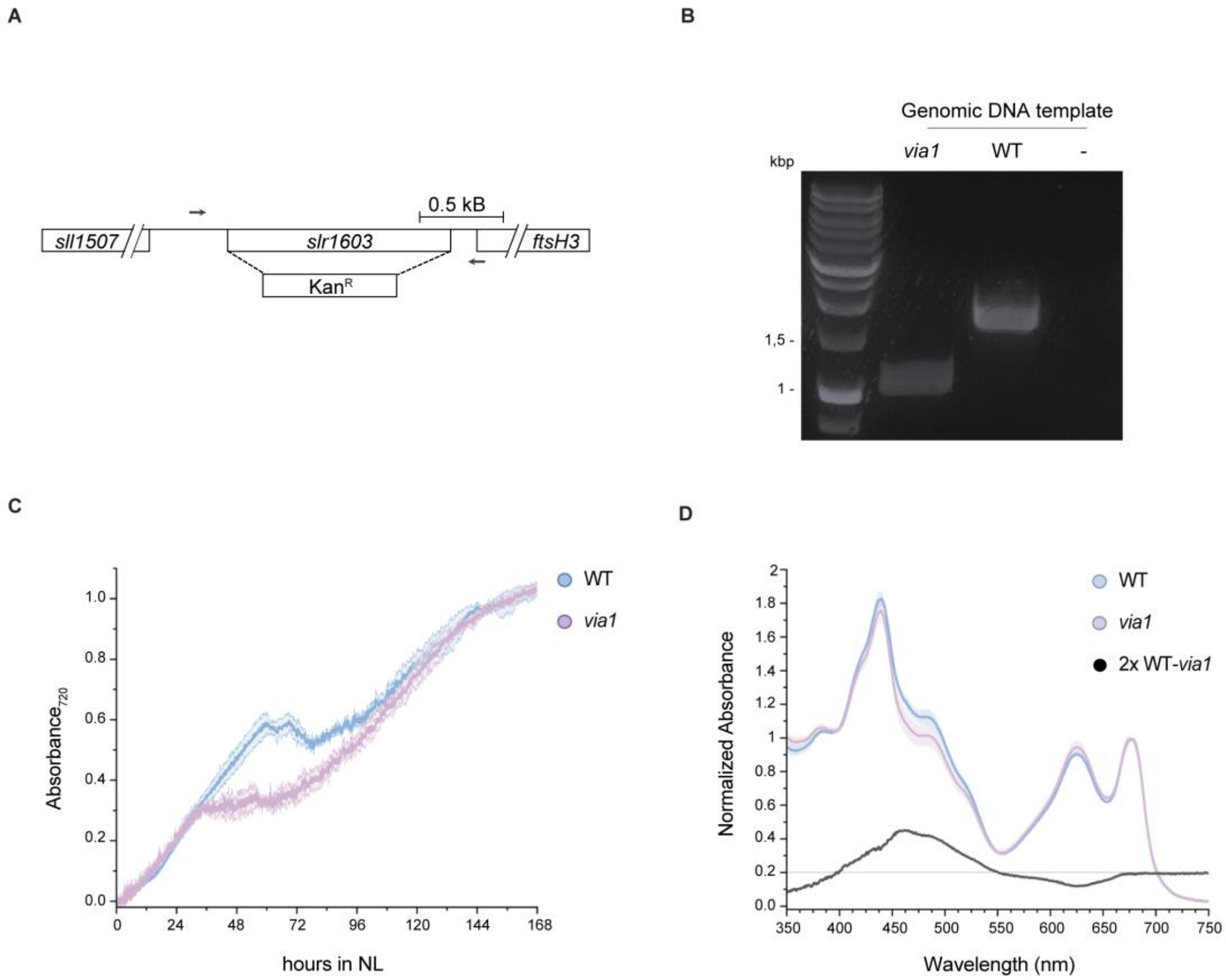

Figure S12. Generation and characterization of a *via1* mutant strain in *Synechocystis sp.* PCC 6803. (A) The entire coding sequence of *VIA1* (*slr1603*) was replaced by a kanamycin resistance cassette (Kan^R) to generate an insertional null mutant. Arrows indicate the positions of the genotyping primers used for PCR analysis in panel B (oYM472-473 in Table S5). **(B)** Successful disruption of the VIA1 locus was verified by PCR analysis of the obtained mutant strain. **(C)** Growth analysis of *via1* mutant grown under normal light conditions (NL; 80 µmol photons m^{-2} s^{-1}). Data represent the mean optical density at 720 nm (OD_{720}) of four independent biological replicates, with shading indicating ± SE. **(D)** Whole-cell absorption spectra of the same cultures. Spectra are normalized to the chlorophyll a absorption peak at 676 nm. The difference spectrum (multiplied by two) between WT and *via1,* which indicates a reduction in cellular carotene levels in *via*1 cells, is shown below as a black line centered at 0.2. Shaded regions indicate standard deviation.

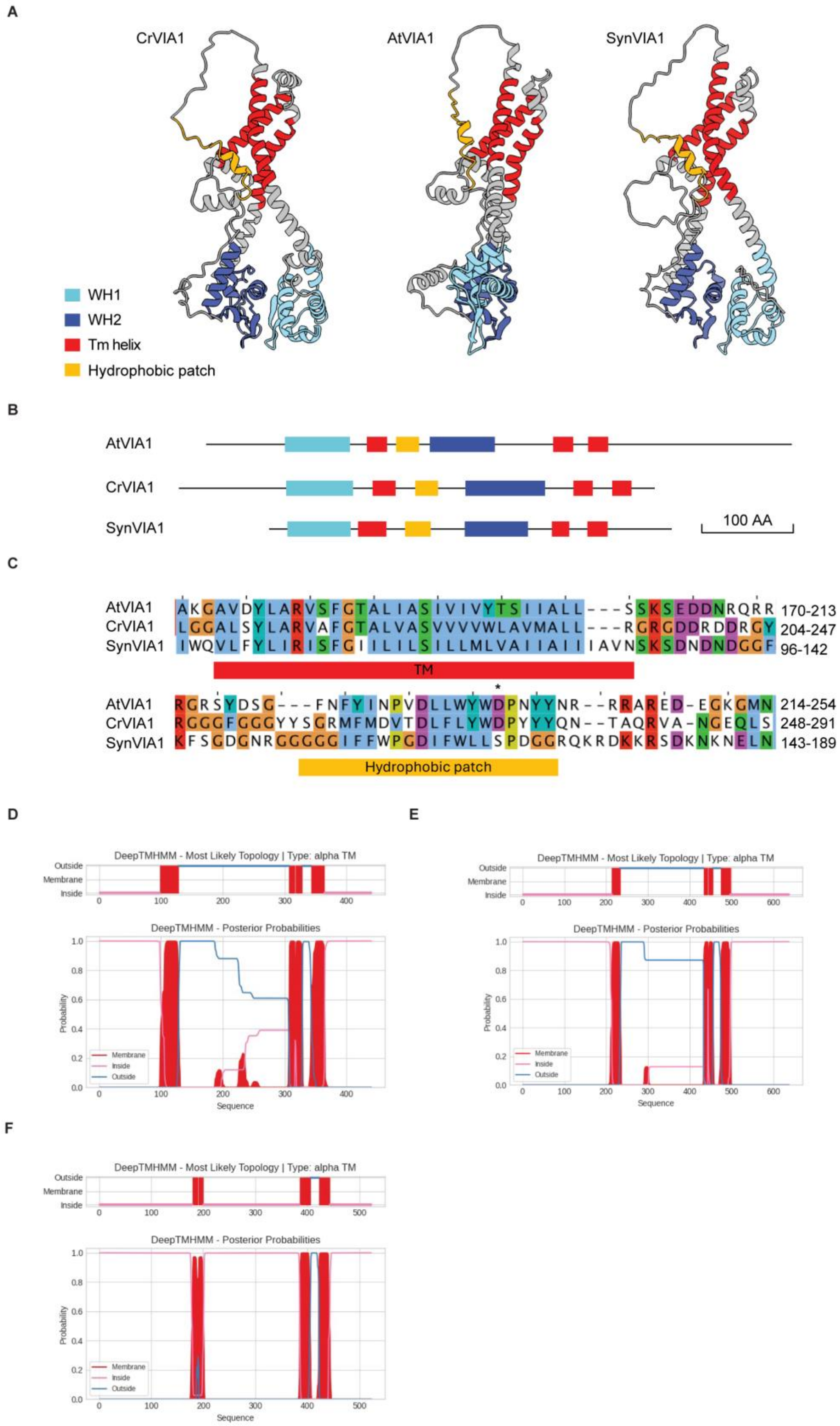

A
CrVIA1
AtVIA1
SynVIA1
WH1
WH2
Tm helix
Hydrophobic patch
B
AtVIA1
CrVIA1
SynVIA1
100 AA
C
AtVIA1 AKGAVDYLARVSFGTALIASIVIVYTSIIALL---SSKSEDDNRQRR 170-213
CrVIA1 LGGALSYLARVAFGTALVASVVVVWLAVMALL---RGRGDDRDDRGY 204-247
SynVIA1 IWQVLFYLIRISFGIILILSILLMLVAIIAIIIAVNSKSDNDNDGGF 96-142
TM
AtVIA1 RGRSYDSG---FNFYINPVDLLWYWDPNYYNR--RRARED-EGKGMN 214-254
CrVIA1 RGGGFGGGYYSGRMFMDVTDLFLYWDPYYYQN--TAQRVA-NGEQLS 248-291
SynVIA1 KFSGDGNRGGGGGIFFWPGDIFWLLSPDGGRQKRDKKRSDKNKNELN 143-189
Hydrophobic patch
D
DeepTMHMM - Most Likely Topology | Type: alpha TM
Outside
Membrane
Inside
DeepTMHMM - Posterior Probabilities
Probability
Sequence
Membrane
Inside
Outside
E
DeepTMHMM - Most Likely Topology | Type: alpha TM
DeepTMHMM - Posterior Probabilities
F
DeepTMHMM - Most Likely Topology | Type: alpha TM
DeepTMHMM - Posterior Probabilities

Figure S13. A conserved hydrophobic stretch in VIA1 may form an additional membrane-spanning segment. (A) AlphaFold3 models of three VIA1 orthologs showing the two WH domains in close apposition. Domain coloring is consistent across panels B and C. **(B)** The domain organization of AtVIA1, CrVIA1, and SynVIA1 includes a single predicted transmembrane helix between WH1 and WH2, which is inconsistent with both WH domains residing on the same side of the membrane. **(C)** Sequence alignment of the three VIA1 orthologs reveals a conserved hydrophobic stretch immediately following the first transmembrane helix, interrupted by a small number of charged residues. **(D–E)** DeepTMHMM predictions for SynVIA1 **(D)** and CrVIA1 **(E)** assign a non-zero probability to a transmembrane helix in this region. **(F)** The corresponding DeepTMHMM prediction for AtVIA1 assigns zero probability, possibly due to an additional aspartate residue that disrupts the continuity of hydrophobic residues (marked with asterisks in panel C).

Table S1. List of reagents used in this study

Reagent type	Designation	Source	Identifiers	Additional information
BG11	17.6 mM $NaNO_3$	Fisher	BP360-500	Recipe from UTEX
	0.23 mM K_2HPO_4	Sigma-Aldrich	P 3786	
	0.3 mM $MgSO_4 \cdot 7H_2O$	Sigma-Aldrich	230391	
	0.24 mM $CaCl_2 \cdot 2H_2O$	Sigma-Aldrich	C-3881	
	0.031 mM Citric Acid·H_2O	Fisher	A 104	
	0.021 mM Ferric Ammonium Citrate	Sigma-Aldrich	F5879	
	0.0027 mM $Na_2EDTA2H_2O$	Sigma-Aldrich	ED255	
	0.19 mM Na_2CO_3	Baker	3604	
	1 mM Sodium Thiosulfate Pentahydrate (agar media only, sterile)	Baker	3946	
	1 mL/L BG-11 Trace Metals Solution	-	-	
BG-11 Trace Metals Solution	46mM H_3BO_3	Baker	0084	Recipe from UTEX
	9 mM $MnCl_2 \cdot 4H_2O$	Baker	2540	
	0.77 mM $ZnSO_4 \cdot 7H_2O$	Sigma-Aldrich	0251	
	1.6 mM $Na_2MoO_4 \cdot 2H_2O$	J.T. Baker	3764	
	0.3 mM $CuSO_4 \cdot 5H_2O$	MCIB	3M11	
	0.17 mM $Co(NO_3)_2 \cdot 6H_2O$	ACROS	10026-22-9	

Gene (*C. reinhardtii*)	*VIPP1*	Phytozome	Cre13.g583550	https://phytozome-next.jgi.doe.gov
Gene (*C. reinhardtii*)	*VIA1, CPLD50, VPL3*	Phytozome	Cre07.g338350	https://phytozome-next.jgi.doe.gov
Antibody	anti-VIPP1 (rabbit polyclonal)	*Gift from Prof. Jean-David Rochaix*		(1:20000)
Antibody	anti-VIPP2 (rabbit polyclonal)	Perlaza et al. 2019		(1:3000)
Antibody	anti-HSP90 (rabbit polyclonal)	Agrisera	AS06 174	
Antibody	anti-α-tubulin (mouse, monoclonal)	Sigma-Aldrich	T6074	(1:10000)
Antibody	anti-ClpP1 (rabbit polyclonal)	*Gift from Dr. Olivier Vallon*		(1:5000)
Antibody	anti-PsaA (rabbit polyclonal)	*Gift from Prof. Jean-David Rochaix*		(1:10000)
Antibody	anti-D1 (rabbit polyclonal)	*Gift from Prof. Jean-David Rochaix*		(1:3000)
Antibody	anti-D2 (rabbit polyclonal)	*Gift from Prof. Jean-David Rochaix*		(1:3000)
Antibody	Anti-CP29	*Gift from Prof. Jean-David Rochaix*		(1:10000)
Antibody	Anti-TIC20	Ramundo et al. 2020		(1:10000)
Antibody	Anti-Ef-Tu	Ramundo et al. 2013		(1:10000)
Antibody	HRP-conjugated anti-rabbit	Promega	W4011	(1:10000)

Antibody	HRP-conjugated anti-mouse	Promega	W4021	(1:10000)
Antibody	anti-FLAG (mouse, monoclonal)	Sigma-Aldrich	F1804	(1:1000)
Antibody	Anti-VIA1 polyclonal	Generated during this study against the winged-helix domains of CrVIA1 ((for details, see Materials and Methods)		(1:20000)
Antibody	OctA-Probe Antibody (anti FLAG)	Santa Cruz Biotechnology	(H-5): sc-166355	(1:2000)
Commercial kit	KOD Hot Start DNA Polymerase	EMD Millipore	71086-5	
Commercial kit	Phusion High-Fidelity DNA Polymerase	ThermoFisher Scientific	F530L	
Commercial kit	Direct-zol DNA Miniprep Plus	Zymo Research	D4019	
Commercial kit	Zymoclean Gel DNA Recovery Kit	Zymo Research	D4001	
Commercial kit	In-Fusion HD cloning plus	Takara	38910	
Commercial kit	SuperSignal West Femto	ThermoFisher Scientific	34095	
Commercial kit	SuperSignal West Dura	ThermoFisher Scientific	34075	
Commercial kit	Protease Inhibitors EDTA-free	Roche	11873580001	
Commercial kit	Dynabeads™ Protein A	Invitrogen	10002D	
Commercial kit	T4 DNA Ligase	New England BioLabs	M0202S	

Commercial kit	NEBuilder® HiFi DNA Assembly Master Mix	New England BioLabs	E2621S	
Restriction enzyme	BsaI	New England BioLabs	R3733L	
Restriction enzyme	SapI	New England BioLabs	R0569L	
Chemical compound, drug	Digitonin	EMD Millipore	300410-5GM	
Chemical compound, drug	Spectinomycin	Sigma-Aldrich	S4014-5G	
Chemical compound, drug	Lugol's Solution	Gatt-Koller	403096459	
Chemical compound, drug	Maxima First Strand cDNA	ThermoFisher Scientific	K1641	
Chemical compound, drug	Fast Start Essential DNA Green Master	Roche	06402712001	
Chemical compound, drug	Glutathione Sepharose 4B resin	Cytiva	GE17-0756-01	
Chemical compound, drug	Bicinchoninic Acid (BCA)	Sigma-Aldrich-Aldrich	1003828215	
Chemical compound, drug	Colloidal Comassie	Molecular Biology Service (MBS, VBCF)		
Chemical compound, drug	3XFLAG	Peptide Facility, VBC	DYKDHDGDYKDHDIDYKDDDDK	Synthesized in-house
Equipment	MagnaRack	Invitrogen	CS15000	
Equipment	Kontes Duall #21 homogeniser	Kimble	8854500021	
Equipment	Mixer Mill MM400, Grinding Jars (50ml) and Grinding Balls (20mm)	Retsch	70354	https://www.retsch.com/products/milling/mixer-mills/
Equipment	Dissection microscope (SporePlay+)	Singer	https://www.singerinstruments.com/solution/sporeplay/	

Equipment	Magnetic stand MagnaRack	Invitrogen	CS15000	
Equipment	Plant LED Grow Light	Phlizon	Cob Series 3000W	
Equipment	Imaging System	iBright CL1500	A44114	
Equipment	Nitrocellulose Blotting Membrane	Amersham	10600001	
Equipment	Scalpel	Aesculap AG	BA821SU	
Equipment	Razor Blade	Apollo Herkenrath	10-210-090	
Equipment	Electron Microscope	FEI Morgagni 268D		FEI, Eindhoven, The Netherlands
Equipment	Multi-Cultivator MC	Photon Systems Instruments	1000-OD	
Software	Sequence Data Analysis	Snapgene		
Software	Statistic Data Analysis	Graphpad		
Software	Molecular Visualization Program	ChimeraX		
Software	Protein Structure Prediction	Alphafold3 Google CoLab		
Software	Protein structure alignment	RCSB PDB		https://www.rcsb.org/alignment
Software	DeepTMHMM	Technical University of Denmark		https://dtu.biolib.com/DeepTMHMM/
Software	Protein Structure Analysis	Alpha Bridge		
Software	Protein Structure Analysis	PICKLUSTER		
Software	Protein Localization Prediction	WoLF PSORT		

Software	Protein Localization Prediction	TargetP 2.0		
Software	Molecular Visualization and Structural Analysis	PyMOL Molecular Graphics System v1.8		Schrödinger, LLC
Software	Conversion of Proteomic Data into Excel Files	MS2Go	https://ms.imp.ac.at/?action=ms2go	Proteomics Facility VBCF

Table S2. List of all the strains used in this study

ID	Strain name	Organism	Description	Comments	Reference
CrRS166	CC-5325	*C. reinhardtii*	wild-type strain	This strain was employed for all the experiments except for the backcross.	
CrRS197	XF305 F1	*C. reinhardtii*	wild-type like progeny from backcross of a CC-5325 strain to a CC-124 strain	This strain was only employed for the backcross experiment.	
CrRS432	*via1-1*	*C. reinhardtii*	(Available at the Chlamydomona s Resource Center)		Cre07.g338350 mutant allele<br><br>Clip ID: LMJ.RY0402.176599
CrRS433	*via1-2*	*C. reinhardtii*	(Available at the Chlamydomona s Resource Center)		Cre07.g338350 mutant allele<br><br>Clip ID: LMJ.RY0402.193812
Cr459	*mars1*	*C. reinhardtii*	*MARS1* (Cre16.g692228) insertional mutant		Cre16.g692228 mutant allele<br><br>Clip ID: LMJ.RY0402.195536<br><br>Perlaza et al. 2019
SPV54.1	T1 to T8	*C. reinhardtii*	Plate containing tetrads obtained from backcross of CrRS432 to CrRS197		Strain generated during this study
SPV54.2	T1 to T10	*C. reinhardtii*	Plate containing tetrads obtained from backcross of CrSR433 to CrRS197		Strain generated during this study

Cr528	*via1-1* + *VIA1*	*C. reinhardtii*	*via1-1* expressing VIA1-3XFLAG	Strain employed for the FLAG IP	Strain generated during this study
A1 SLM3	*via1-1* + *mutVIA1-1*	*C. reinhardtii*	*via1-1* expressing mutVIA1; mutVIA1 contains the following point mutations A70D S71A G72A E285Q A285D G286V	Strain employed for the VIPP1 IP and the HL growth assay	Strain generated during this study
A3 SLM3	*via1-1* + *mutVIA1-2*	*C. reinhardtii*	*via1-1* expressing mutVIA1; mutVIA1 contains the following point mutations A70D S71A G72A E285Q A285D G286V	Strain employed for the HL growth assay	Strain generated during this study
A3 SLM2	*via1-1* + *AtVIA1-1*	*C. reinhardtii*	*via1-1* expressing AtVIA1, the ortholog of VIA1 from *Arabidopdis thaliana*	Strain employed for the HL growth assay	Strain generated during this study
A11 SLM1	*via1-1* + *SynVIA1-1*	*C. reinhardtii*	*via1-1* expressing SynVIA1, the ortholog of VIA1 from *Synechocystis sp pcc 6803*	Strain employed for the HL growth assay	Strain generated during this study
261	SynVIA1-FLAG	*E. coli*	DH5a transformed with SynVIA1-FLAG plasmid	Verified by restriction digest and sequencing. CM^R resistant	

263	ΔSynVIA1	*E. coli*	DH5a transformed with ΔSynVIA1 deletion plasmid	Verified by restriction digest and sequencing. CM^R resistant	
272	SynVIA1-FLAG	*Synechocystis sp.* PCC *6803*	Syn transformed with SynVIA1-FLAG plasmid	Verified by restriction digest. CM^R resistant	
276	ΔSynVIA1	*Synechocystis sp.* PCC *6803*	Syn transformed with ΔSynVIA1 deletion plasmid	Verified by restriction digest, PCR, and sequencing. Kan^R resistant.	

Table S3. List of all plasmids used in this study

Plasmid	Gene	Purpose	Source
pRAM254	*pVIA1:VIA1-NeonGreen-3×FLAG*	*via1-1* mutant complementation	Plasmid generated during this study
pRAM315	*pVIA1:VIA1-3×FLAG*	*via1-1* mutant complementation	Plasmid generated during this study
pRAM321	*VIA1_A70D|S71A|G72A|E285|QA285D|G286V (gene ordered from Twist Bioscience)*	*via1-1* mutant complementation	Plasmid generated during this study
pRAM324	*AtVIA1 (gene* ordered from Twist Bioscience)	*via1-1* mutant complementation	Plasmid generated during this study
pRAM537	*SynVIA1 (*gene ordered from Twist Bioscience)	*via1-1* mutant complementation	Plasmid generated during this study
pNS001	Empty vector	Backbone for cloning pRAM254 and pRAM315	*Gift from Masayuki Onishi*
pCSB192	Empty Level 0 and 2	Cloning of pRAM321, 324, and 537	*Gift from Pawel Mordaka*
pStA0_16S	rrn16S promoter and 5'UTR	Cloning of pRAM321, 324, and 537	*Gift from Pawel Mordaka*
pStA0_TrbcL	*RbcL* terminator and 3'UTR	Cloning of pRAM321, 324, and 537	*Gift from Pawel Mordaka*
pGT402	Empty Level 1	Cloning of pRAM321, 324, and 537	*Gift from Pawel Mordaka*
pHT150	*psbH* right flanking sequence	Cloning of pRAM321, 324, and 537	*Gift from Pawel Mordaka*

pHT146	*psbH* left flanking sequence	Cloning of pRAM321, 324, and 537	*Gift from Pawel Mordaka*
SynVIA1-FLAG	3x FLAG tag motif with flag linker to C-term of SynVIA1 gene	Co-Immunoprecipitation	Generated by the Mazor lab
ΔSynVIA1	replacement of *SynVIA1* gene with Kan^{RR} gene	Growth phenotype	Generated by the Mazor lab
pJET1.2	Plasmid containing Kan^{RR} gene	Used to clone Kan^{RR} via PCR	Thermo Scientific

Table S4. List of Twist fragments/vectors used in this study

Name	Gene	Sequence of the insert	Purpose
69	*AtVIA1*	aaggggttggtctcatgtggctcttcgatggcaagccttgacaaagtctccg<br>gtgcgattaagcccggcggtttagtcgaatctgataaattacccaccgacgt<br>tcgtaagcgggccatggacgccgttgacgaatgcggacgtcgtgtgacggt<br>gggagacgtagcgtcacgaggtggattaaaagtgacggaagcgcaaact<br>gccctgcaggccattgcagccgacacagacggcttccttgaagtcagtgac<br>gagggagacgtcttgtacgtgtttcctcgagattaccggacgaaactggca<br>gctaaatccctcagaatccagatagagccttttctcgagaaagccaaaggg<br>gccgtggactacctggcacgggtgtcgttcggaaccgctttaatcgcttcga<br>ttgtgatcgtttatactagtataatcgcattgttatcctctaagtccgaagac<br>gacaacagacagcgccgccggggacgtagctacgactccggttttaacttt<br>tacataaatccagtagaccttctgtggtattgggacccgaactactataatc<br>gcagacgtgcgcgtgaggacgagggcaaaggtatgaactttatcgagagc<br>gtgtttagtttcgttttcggggacggggatccgaaccagggcattgaagagg<br>aacgatggcaaatgatcggccaatacattacatcccgaggcggggtggtg<br>gcagcagacgagctggcgccgtatctggacgttcctagcagtaaatcagcg<br>atgaatgacgagtcgtatatcctgcccgtactcctgcgtttcgacggacagc<br>cagaattagacgaggaaggtaacatcctttactgcttcccaagtttgcagcg<br>gactgcgagcggcagctcccgtagaaaggagtacgttgggaagtggttcg<br>attgggtcgctgacatggaaaagttctttaaagaaaagaagtggcagttct<br>ccaagacgtctacgtcggaacgtgccctcgtaataggtttaggggctgtca<br>accttttcggggtgatagtgcttaatactctgcttaatgaaatgagtgtacgt<br>ccgggtggcttcctgaccttcgtaaagaatatctaccctctgttacaaattta<br>tgccgggagtttcttcacgattccacttatacgttggttctctattaaacgga<br>agaacaaccaaattgaaaatcgtaataaggcccgtctgcagttcgcccgtg<br>ccttagagtcaccggacattgccctgcgccggaaactgctgtcagcgcgtg<br>acatggcccagaagactgtaattggcaaagacagaattgtttatagcaccg<br>accgtgacatgatggagcagaattacgaaaccgatgagtgggatcgccgc<br>tttaaggaactggagaagtcagactaatgaagagcgaccagagacccaac<br>actg	Generating pRAM324
pRAM418	*SynVIA1*	aaggagatatacatatgcaccaccaccaccaccattcgagtggcctggaa<br>gtgctgttccagggacctggttccatgatcgttgcgagcgaagggagcggt	Generating pRAM537

		atgagcgtggcgttcaacccgcagatcatgactgccgtcgaacaattagat tacgtggttacagttggcgatgttgcgagtcaagccggcctcgagatcaatc agacacagcaaggcctcttgaccctggccagcgaagtcgaaggtcacttac aagtcgcagaatcaggtgagatcgtattcgcgttccctaaacaatttcgcac catcctgcgtaacaaatactggcgcctgcgctttcaatcttggcttcagtcta tctggcaagttctgttctatcttattcgtatctcgttcggaatcattctgattct gtcgattctcttaatgctggttgcaattatcgcgatcattattgctgtgaactc taaaagtgacaatgacaatgacggcggcttcaagttctcgggcgatggcaa tcgcggcggcggcggcggtattttcttttggcctggcgatatcttttggctgtt gtcgccggacggcggtcgccagaagcgtgacaagaagcgctcagacaag aacaagaatgaactgaactttctggaagcaatctttagcttcctgttcggtg atggcaatcctaacgaagaccttgaggagaagcgttggcagactttggggt ccctgatccgtcacaatggtggtgcaattgcagcggagcaagctgcaccat acctggacaacgtcactaagtttaaccgcgataacgaagattatattatccc ggttttggctcgcttcaacggctacccacaggtaagcccgagtggggagttg atctatacgtttcctaatcttcaggtatcagcgcaagaacgtcagagcacta ccctgtcagcgtatttacgtgagaagccgtggaagttcagtcaggcctcgtc aggtcagaagattgctgcaattgcacttggcggcgtcaatctgatcctcgcg ctctctctgggagtgatgttaaagacgtatggtgccgaactggacctcggca ctttagtgtactttgtgcaatcaatctatggtgtgttggtcacgtacgccattg ggttcttagccatcccgctggtacgttacttttggttacaggagcgtaaccag aagttggagcagcgtaatggggaccgccagcaacgtgcaaagctgctcca gcccccgagtcccccgctgaaggccaagctggagttcgcgcaagaatggg caacgtcgacggttatcacgcctgccgatattacatatcgtacggatcagga ttcgttagaacaagaattcaagcgtttcacaccgacgcaaggcgattgaag cagcggcttagaagttttgtttcaaggtccaggatcc	
48	*VIA1_A70D|S71A |G72A|E285|QA2 85D|G286V*	aaggggttggtctcatgtggctcttcgatggtagaattggtcaagagagaa gcgggccttcaatcaaaccgccttgaacctgatctgagagaacgtgtggaa tcggcgatagagcgcttggggggcagagtcaccgtgggagacgtggccgc tagagctggggtgaagcttgctcaagcagacgaagccttaaaagctctggc gtacgacaccgccgcagcgcttcaggtatctgacgcagcagatcttgtctac gcctttgccccggattttagatcgcgtttgcggtcgcggtcactgttagtttct acactgttaccccctgggcagacgcctgggaggcgcccttagttacttagcgc gggtcgcgttcggaacagcgttggtagcaagtgtagtagttgtttggttagc cgtcatggccctgctgcgtgggagaggtgacgaccgcgacgatcgtggata tcggggcggagggttcgggggtggctattattctggccgcatgttcatggat gttaccgatcttttcttatactgggatccctattactatcagaacaccgctca acgcgtcgccaacggcgaacaattgtcgttcgtcgagtctattttttccttcg tttttggagatggggacccgaacgcagacttcgaagagcgccgctggcaac gccttggggaaatgatacgtgcgaagggcggtgtggtcacggcggaagaa atggcgccgtacttagaccctccggaacctgctgacccgccgggtcgtcca ctttacgacagttcaggaatgacagaaacttacattccttatcctgacgaaa acttcgtcctgccggcgttaattaaatttggtggggaaccgagcgtggatca agacgtaagaatcttgtatcgtttccctgccctgcagttaactggtgtcaagg gcaaacgcccagctaatcgcttttcacctcagtcgtcattcgaggttccaat ggaacgggattggcagttcaccgcagcttctggtgggcaagtcgctggaac tgttttcttgggtctgttaaatttagtaggggtagcagtcttatcgtccttaat ggctgatccacgtgccgtgtacgtgctggccgcgcaagggctggggtttgtc ccagggctgttgggtccgttacagctgtatgcagccgctttttcgccatccc cgcattgagatggttaatcaactcttccagaaatagacaaattgatgcgcg	Generating pRAM321

		caacgcagcccgggccgccgctgccggtcttctgaatggtccagcggccgt<br>tgcgggctgggtggcggacaaattggccgcggcccgtcagttgggttcttcc<br>agcagcgcatctgctagtaatgctaatttggtcgatcggccgtttgagcggc<br>agttggctccccctcgcctgggaccgcggcttgttggagacgacgatgtggt<br>ctacgattccgggagagaggcggaaccccagttgcttgaaagagacttgg<br>acgattgggactcccggttgagtggaagagaagcggggggtggaggggcc<br>ggcgctcgcagagcggggggtggcggtggaacgagaggctctggaagtg<br>ggaatggaggagtggctagaggcggtcgttttcgcagataatgaagagcg<br>accagagacccaacactg	

Table S5. List of oligonucleotides used in this study

Primer name	Sequence 5' → 3'	Purpose
oRS01	CCGAGGAGAAACTGGCCTT	Genotyping
oRS30	GCACACGCTAACATCTACAA	Genotyping
oRS37	GCTTCGTGGAGTCCATCTTC	Genotyping
oRS38	CCTAGTCCACCCTCACCGTA	Genotyping
oRS39	TACGGTGAGGGTGGACTAGG	Genotyping
oRS40	GGTATGAAACCTCTCGCCAA	Genotyping
oRS41	TCTGGGCCCACTTGGTCTTGAGC	Amplifying *CrVIA1* from gDNA
oRS42	AGGTACGGCCTGTGCTGCTTACTGACT	Amplifying *CrVIA1* from gDNA
oRS43	AAGCAGCACAGGCCGTACCTTTC	Amplifying *CrVIA1* from gDNA
oRS44	TACCTCACCCTACCGTCCCCGTCGGAACC	Amplifying *CrVIA1* from gDNA
oRS47	TGTTCATGGACGTGACGGAC	RT-qPCR_ *CrVIA1*_A_Fw
oRS48	CCCTTGGCTCGGATCATCTC	RT-qPCR_ *CrVIA1*_A_Rv
oRS57	GTCTGTGTGTGTTGTGCAATGGG	RT-qPCR_ *CrVIA1*_B_Fw
oRS58	GCTCTCTCTCGTGTCACCTTTG	RT-qPCR_ *CrVIA1*_B_Rv
oRS59	ATCTTGTGGACCGGCCTTTT	RT-qPCR_ *CrVIA1*_C_Fw
oRS60	GTCGTACACCACGTCATCGT	RT-qPCR _ *CrVIA1*_C_Rv
oRS61	GACGATGACGTGGTGTACGA	RT-qPCR_ *CrVIA1*_D_Fw
oRS62	ACTGTCCCAGTCGTCCAGAT	RT-qPCR _ *CrVIA1*_D_Rv
oRS822	CAAGTACACCATTGGCGAGC	RT-qPCR_*GBLP*_Fw
oRS823	CTTGCAGTTGGTCAGGTTCC	RT-qPCR_*GBLP*_Rv
oRS401	CCGGTTCCGACGGGGATCCGGCGGTGGCGACTA	Generating pRAM315 Infusion
oRS402	GTCCTTACTTGTCGTCATCGTCCTTGTAGTCGA	Generating pRAM315 Infusion

oRS403	GACAAGTAAGGACGGTAGGGTGAGGTAGGT	Generating pRAM315 Infusion
oRS404	GAAGCTTGATATCAGCGGCCGTCCATTACAT	Generating pRAM315 Infusion
oRS405	GATATCAAGCTTCTTTCTTGCGCTATGAC	Generating pRAM315 Infusion
oRS406	CCGTCGGAACCGGC	Generating pRAM315 Infusion
oRS1602	GGGGAACCGAGCGTGGATCAAGACGUAAGAATCTTGT	Generating pRAM321 Mutagenesis from pRAM323
oRS1603	ATCCACGCTCGGTTCC	Generating pRAM321 Mutagenesis from pRAM323
oRS1604	CGCAGCGCTTCAGGTCTCTGACGCAGCAGATCTTGTCTA	Generating pRAM321 Mutagenesis from pRAM323
oRS1605	CGCTGCGGCGGTGTCGTA	Generating pRAM321 Mutagenesis from pRAM323
oRS1852	AAAGAGACTTGGACGATTGGG	RT-qPCR to check At*VIA1* expression
oRS1853	CCATTCCCACTTCCAGAGC	RT-qPCR to check At*VIA1* expression
oRS1856	CAAGCAGACGAAGCCTTAAAAGAAAGAGACTTGGACGATTGGG	RT-qPCR to check *mutVIA1* expression
oRS1857	AGTGTAGAAACTAACAGTGACCGCCATTCCCACTTCCAGAGC	RT-qPCR to check *mutVIA1* expression
oRS1864	CCTTTTGGAAACTTCGGCTTCCAAGCAGACGAAGCCTTAAAAG	RT-qPCR to check *SynVIA1* expression
oRS1865	CGCTGCCATTCTCCAAATTGAGTGTAGAAACTAACAGTGACCG	RT-qPCR to check *SynVIA1* expression
oYM441	GGAGTGGAAACCACCGTTACCATGTCATGATAATAATG	*SynVIA1-FLAG* backbone reverse
oYM442	CATGACATGGTAACGGTGGTTTCCACTCCAG	*SynVIA1-FLAG* |upstream forward

oYM443	CTAAGGATAGAGGCTTTGTAACTGTTGGTCAACTGTCACTTG TCGTCGTCGTCCTTGTAGTCGATGTCGTGGTCCTTGTAGTCA CCGTCGTGGTCCTTGTAGTCAAATTCGCCGGAGCCGGCGGC GGAGCCGGCGGAGCCGTCCCCCTGGGTTGGGGTAAAGCGT TTAAACTCCT	*SynVIA1-FLAG* | 3xFLAG and linker reverse
oYM444	GCCTCTATCCTTAGCATTTTTTCCGAATAAATACCTGTG	*SynVIA1-FLAG* | FLAG and linker forward
oYM445	GGTCAACTGCCCAGGCGTTTAAGGGCACC	*SynVIA1-FLAG* |CM^R reverse
oYM446	CGCCTGGGCAGTTGACCAACAGTTACAAAG	*SynVIA1-FLAG* | down forward
oYM447	GTGGGATTCTACCAATTGCTACTGG	*SynVIA1-FLAG* | down reverse
oYM448	CAATTGGTAGAATCCCACAATTCCACACATTATAC	*SynVIA1-FLAG* | backbone forward
oYM492	GATGAGTTTTTCTAACAGTTGACCAACAGT	ΔSynVIA1_Kan^R | backbone forward
oYM493	CCCGTTGAATATGGCTCATTAAAAATTATTCTTC	ΔSynVIA1_Kan^R | backbone reverse
oYM494	GAAGAATAATTTTTAATGAGCCATATTCAACGGG	ΔSynVIA1_Kan^R | backbone reverse
oYM527	GAAGAATAATTTTTAATGAGCCATATTCAACGGG	ΔSynVIA1_Kan^R | Kan^R forward
oYM528	ACTGTTGGTCAACTGTTAGAAAAACTCATC	ΔSynVIA1_Kan^R | Kan^R reverse
oYM472	GCACCAGCCAACAGGCTAGCAAACATT	Forward primer used for verification of *slr1603* gene replacement
oYM473	TCCACCGGTTAGCAATGGATGGAGTGGATAA	Reverse primer used for verification of *slr1603* gene replacement

Material and methods

Growth assay (*Synechocystis sp.* pcc 6803)

Cells were initially grown for 14 days in 50 mL flasks of BG11 supplemented with 6 µg/ml Ferric ammonium citrate. Cultures were then diluted into 500 ml flasks and acclimated to low light intensity (8 µmol photons m^{-2} sec^{-1}) for three days before inoculation into the bioreactor (Multi-

Cultivator MC 1000-OD, Photon Systems Instruments). After this acclimation period, each culture was split into four 50mL subcultures, which were used to initiate growth measurements in the bioreactor. Light intensity was set to either normal light (80 µmol photons m^{-2} sec^{-1}) or high light (200 µmol photons m^{-2} sec^{-1}), and absorbance at both 720 nm and 680 nm was recorded every 10 minutes. Cultures were bubbled with air throughout growth. Fluorescence emission spectra were recorded on a Fluoromax-4 spectrofluorometer (HORIBA Jobin-Yvon). For 77 K measurements, slit width of 5 nm and 3 nm were used for the entrance and exit monochromators, respectively. Samples were diluted to an OD_{680} 0.1 in a final glycerol concentration of 40%, frozen around thin glass rods in liquid nitrogen, and excited at 440 nm. Whole cell absorbance was measured after 180 h of bioreactor growth using a Cary 4,000 UV–Vis spectrophotometer (Agilent Technologies) equipped with an integration sphere.

Isolation and genotyping of *C. reinhardtii VIA1* insertion strains

Genomic DNA extraction from WT, *via1-1,* and *via1-2* liquid cultures in the exponential growth phase and in standard light conditions (~80 µmol photons m^{-2} s^{-1}) was carried out as previously described[1]. The insertion of the mutagenic cassette in the *VIA1* locus was verified by PCR in each mutant. All PCRs were performed using KOD Hot Start DNA Polymerase (EMD Millipore). In the *via-1* strain, the insertion occurs in exon 10. Therefore, primer pairs oRS39 and oRS40 were designed to anneal in exon 9 and the 3'UTR, respectively (see Fig. S2 for details). This primer pair allows the amplification of the WT locus as well as of the mutated locus containing the cassette (PCR conditions: 95 °C 2 min, 95 °C 20 sec, 55 °C 10 sec, 70 °C 1 min 40 sec). To selectively amplify a section of the cassette inserted in the *CrVIA1* locus, the oRS01 primer was designed to anneal within the mutagenic cassette and was used in conjunction with oRS39 (PCR conditions: 95 °C 2 min, 95 °C 20 sec, 55 °C 10 sec, 70 °C 28 sec). For *via-2*, which harbors the insertion within intron 7, primer pairs oRS37 and oRS38 were designed to anneal in exon 6 and exon 8, respectively. This primer pair allows the amplification of the WT locus as well as of the mutated locus containing the mutagenic cassette (PCR conditions: 95 °C 2 min, 95 °C 20 sec, 55 °C 10 sec, 70 °C 1 min 40 sec). Moreover, to selectively amplify a section of the cassette inserted in the *CrVIA1* locus, the oRS30 primer was designed to anneal within the mutagenic cassette and was used in conjunction with oRS38 (PCR conditions: 95 °C 2 min, 95 °C 20 sec, 55 °C 10 sec, 70 °C 30 sec). To ensure the quality of the employed genomic DNA (gDNA), a mating type-specific PCR[2] was performed as a control. All PCR reactions were subjected to electrophoresis on 1% [w/v] agarose gels to verify their expected sizes.

Backcross and tetrad dissection analysis

Cells were streaked onto a fresh TAP agar plate and grown under light (80 µmol photons m^{-2} s^{-1}) for approximately 3-4 days. The cells were subsequently restreaked onto a TAP agar plate containing one-tenth of the standard NH_4Cl concentration to induce nitrogen starvation and gametogenesis. The day after, gametes were suspended in 100 µL of sterile water to a final density

of ~1×10^6 cells mL^{-1} and incubated with gentle agitation for 30-60 minutes under light (80 µmol photons m^{-2} s^{-1}). Mating efficiency was monitored every 30 minutes by mixing 2 µL of the mating mixture with 8 µL of Lugol's solution (Gatt-Koller) and scoring quadriflagellate formation under a microscope. Mating typically occurred within 2 h. Subsequently, 100 µL of the mating mixture was spotted onto the center of a TAP 4% [w/v] agar plate and incubated in the dark (covered with aluminum foil) for 6 days to allow zygote maturation. Vegetative cells were then gently removed by scraping the plate surface with a rectangular razor blade (Apollo Herkenrath), leaving zygotes attached to the agar. To recover zygotes, 100 µL of liquid TAP was added to the plate surface, and zygotes were detached with a different razor blade (Aesculap AG). The suspension was transferred onto several fresh TAP plates containing 1.6% [w/v] agar and spread in a line in the middle of each plate. Plates were then incubated under light (80 µmol photons m^{-2} s^{-1}) for 24 h to induce germination. Tetrads were identified and dissected using a SporePlay+ dissection microscope (Singer Instruments). Once colonies became visible to the naked eye, progeny were re-arrayed into a 96-format grid and replicated onto TAP plates with or without paromomycin for segregation analysis.

Phylogenetics and species distributions

To reconstruct the VIA1 phylogeny, the *C. reinhardtii* ortholog (Cre07.g338350) was searched against a series of predicted proteomes representing eukaryotic and prokaryotic diversity using Diamond BLASTP v2.1.11 (ultra-sensitive, $E < 10^{-5}$)[3]. These datasets comprised predicted chlorophyte proteomes (n = 13), plant proteomes (n = 37), the EukProt v3 TCS dataset, and selected prokaryotic reference proteomes (n = 5,143) from UniProt[4,5]. The resulting hits were extracted and aligned with MAFFT v7.520, and alignments were trimmed with a gap-threshold of 50% using trimAl v1.4[6,7]. Initial phylogenies were inferred using IQ-Tree v2.3.6 and the LG4M substitution model with support derived from Shimodaira-Hasegawa approximate likelihood ratio tests (SH-aLRT, n = 1,000)[8,9]. Orthologs were selected phylogenetically and extracted before being re-aligned with MAFFT using the L-INS-i algorithm. The alignment was trimmed with a gap-threshold of 10% and used to generate a hidden Markov model (HMM) using HMMER v3.1b2[10]. To increase search sensitivity, proteomes were re-searched using the VIA1 HMM ($E < 10^{-5}$) and the resulting hits were curated phylogenetically as described. To generate the final phylogeny, an alignment was generated using VIA1 homologs and MAFFT L-INS-i before being trimmed with a gap-threshold of 50%. The phylogeny was inferred using IQ-Tree and the LG+C60+F+I+R8 substitution model selected using ModelFinder[11]. The phylogeny was visualized using FigTree v1.4 (http://tree.bio.ed.ac.uk/software/figtree/) and ITOL v6[12]. The curated VIA1 homologs were further used to assess the distribution of VIA1 across photosynthetic species. To account for missing gene models, the genomes of taxa lacking VIA1 were searched using MiniProt v0.18, and homologs were predicted and phylogenetically curated when found[13]. Protein sequences, alignments, phylogenies, and datasets are available from here: https://figshare.com/s/c411a207630c1d30d379

Total protein extraction and immunoblot analysis (*C. reinhardtii*)

For all experiments, total protein extracts were prepared from whole-cell lysates under denaturing conditions. Cells from 5 mL cultures in the exponential growth phase were harvested by centrifugation at 3,000 × g for 5 min, and pellets were resuspended in 150 µL SDS lysis buffer (100 mM Tris-HCl, pH 8.0, 600 mM NaCl, 4% [w/v] SDS, 20 mM EDTA) freshly supplemented with EDTA-free protease inhibitor cocktail (Roche). Samples were vortexed for 10 min at room temperature, incubated for 30 min at 37 °C, and clarified by centrifugation at 21,300 × g for 15 min at room temperature to remove insoluble debris.

The supernatant was transferred to a new tube and supplemented with ¼ volume of 5× SDS loading buffer (250 mM Tris-HCl, pH 6.8, 5% [w/v] SDS, 0.025% [w/v] bromophenol blue, 25% [v/v] glycerol), freshly supplemented with 5% [v/v] 2-mercaptoethanol. A 5 µL aliquot of each extract was retained for protein quantification using a bicinchoninic acid (BCA) assay according to the manufacturer's instructions (Sigma-Aldrich). Specifically, 2 µL of protein extract was mixed with 200 µL BCA working solution (1:50 BCA: copper sulfate), incubated for 5 min at 50 °C, and absorbance was measured at 562 nm using a BSA standard curve.

For immunoblotting, 40 µg of total protein per sample was resolved by SDS–PAGE using Criterion precast gels (Bio-Rad) and transferred onto 0.2 µm nitrocellulose membranes (Amersham). Membranes were blocked for 1 h at room temperature in PBS-T supplemented with 5% [w/v] nonfat dry milk (Maresi). Primary and secondary antibodies (listed in Table S1) were diluted in blocking buffer. Primary antibody incubations were performed for 1 h at room temperature, except for anti-D1 and anti-VIPP2, which were incubated overnight at 4 °C. HRP-conjugated anti-rabbit or anti-mouse secondary antibodies (Promega) were applied at 1:10,000 for 1 h at room temperature.

Following each antibody incubation, membranes were washed three times for 10 min in PBS-T containing 1% [w/v] nonfat dry milk. Signals were detected using luminol-based enhanced chemiluminescence (SuperSignal West Dura or West Femto; Thermo Fisher Scientific).

Immunoblot analysis (*Synechocystis sp.* PCC 6803)

For immunoblotting, 100 µg of total protein per sample was resolved by SDS–PAGE using Criterion precast gels (Bio-Rad) and transferred onto 0.45 µm nitrocellulose membranes (GVS #S74181100). Membranes were blocked overnight at 4 °C in PBS-T supplemented with 5% [w/v] nonfat dry milk (Nestle Carnation). Primary antibodies (listed in Table S1) were diluted in blocking buffer. For Flag detection an HRP conjugated OctA-Probe Antibody from Santa Cruz Biotechnology. Signals were detected using luminol-based enhanced chemiluminescence (West Femto; Thermo Fisher Scientific).

Chlorophyll Fluorescence Measurements (*C. reinhardtii*)

Chlorophyll fluorescence measurements of *Chlamydomonas* were performed using a Dual PAM 100 fluorometer (Walz, Germany). Cells were grown in minimum media for 5 days, harvested

during the exponential growth phase and subsequently subjected to a 30-minute period of dark adaptation while maintaining gentle agitation. The following parameters were calculated: Photosystem I Quantum Yield ((Pm - Pm')/Pm), Photosystem II Quantum Yield (Fv/Fm) and Non-Photochemical Quenching ((Fm - Fm')/Fm') where Fm' is the maximal chlorophyll fluorescence yield during exposure to light. Calculations were based on established methodologies for the measurement of photosynthetic parameters[14,15]. New measurements in stress conditions were performed using an AquaPen AP110/C fluorometer (Photon Systems Instruments). Maximum quantum yield of PSII (Fv/Fm) was measured in liquid cultures over a 48-hour growth assay under normal light (80 µmol photons m^{-2} s^{-1}) and high light (800 µmol photons m^{-2} s^{-1}). Experimental conditions, including cell density and chlorophyll concentration at the start of the assay, are described in the "High light assay and time course" section. Fv/Fm (Qy) measurements were performed using the blue measuring light (455 nm) with a 30% flash pulse, a 70% super pulse, and an actinic pulse of 300 µmol photons m^{-2} s^{-1}.

High light assay and time course (*C. reinhardtii*)

Liquid cultures were initiated from freshly streaked colonies (3-4 days old) grown on TAP plates under continuous light (80 µmol photons m^{-2} s^{-1}). Cells were inoculated into 15 mL TAP in 100 mL glass flasks and grown for 2–3 days under continuous light (80 µmol photons m^{-2} s^{-1}) with shaking at 130 rpm. Once cultures reached ~1×10^6 cells mL^{-1}, they were expanded to 50 mL and grown for an additional 1–2 days. Total chlorophyll was quantified by methanol extraction as described previously[17], and cultures were normalized to 8 µg chlorophyll mL^{-1} in a final volume of 31 mL in 50 mL Falcon tubes. Three technical replicates were prepared for each strain. The high light treatment was performed at ∼800 µmol photons m^{-2} s^{-1}, employing a Plant LED Grow Light (Phlizon, Cob Series 3000W) positioned ∼40 cm away from the cultures. Light intensity was measured along the length of each tube to ensure uniform illumination, and tubes were randomly repositioned every 24 h to minimize positional effects. Transfer to HL conditions resulted in a temperature increase of ~4–5 °C.

For HL growth assays, cultures were photographed, and chlorophyll content was measured at the time points indicated for each experiment. For the HL time-course immunoblot experiment, cultures were adjusted to a final volume of 100 mL at the start of HL exposure and illuminated as above for 8 h. 5 mL aliquots were collected at 2-h intervals, pelleted by centrifugation (3,000 × g, 5 min), and stored at –20 °C for subsequent total protein extraction.

Transmission electron microscopy (*C. reinhardtii*)

Wild-type (WT) and *via1-2* cell cultures were subjected to HL (1000 μmol photons m^{-1} s^{-1}) for 0, 2, 4, and 6 h. 10 mL aliquots from each time point, at a concentration of approximately 1×10^6 cells mL^{-1}, were gently spun down at 500 x g for 3 minutes, and the resulting cell pellets were fixed using a mixture of 2% [v/v] paraformaldehyde (Electron Microscopy Sciences, Hatfield, PA) and 2% [v/v] glutaraldehyde (Agar Scientific, Essex, UK) in 0.1 M sodium cacodylate buffer, pH 7.4. This fixation process was carried out overnight at room temperature. Samples were then rinsed

with the same buffer, post-fixed in 2% [w/v] osmium tetroxide (Agar Scientific, Essex, UK) in 0.1 M sodium cacodylate buffer, pH 7.4, dehydrated in a graded series of acetone, and embedded in Agar 100 resin (Agar Scientific, Essex, UK). Subsequently, 70 nm sections were cut and post-stained with 2% uranyl acetate [w/v] and Reynolds lead citrate (Delta Microscopies, Mauressac, France). Micrographs were recorded on a FEI Morgagni 268D (FEI, Eindhoven, The Netherlands) operated at 80 keV, equipped with a Mega View III CCD camera (Olympus-SIS).

Chloroplast subfractionation

Chloroplast subfractionation was performed using a protocol modified from[16]. *via1-1* + VIA1 cultures were grown in liquid TAP under continuous light (80 µmol photons m^{-2} s^{-1}) for 3 days to a density of ~1×10^6 cells mL^{-1}. Cells were harvested by centrifugation and resuspended in an equal volume of 0.33 MS buffer (25 mM MES-NaOH pH 6.5, 1.5 mM NaCl, 0.33 M sucrose) supplemented immediately before use with EDTA-free protease inhibitor cocktail (Roche). Cells were lysed by five freeze–thaw cycles (snap-freezing in liquid nitrogen and thawing at room temperature).

Subcellular fractionation was performed in open-top polyclear ultracentrifuge tubes (SETON Scientific, 7010) containing a two-step sucrose gradient prepared in 0.33 MS buffer (2 mL 1.3 M sucrose over 2 mL 1.8 M sucrose). 200 µL lysate was layered onto the 1.3 M cushion and centrifuged at 139,863 × g for 30 min at 4 °C (SW60 Ti rotor; Beckman Coulter). The supernatant above the 1.3 M cushion (stroma fraction) was collected, transferred to polycarbonate tubes (Beckman Coulter, 343775), and clarified at 150,000 × g for 1 h at 4 °C (TLA-100 rotor; Beckman Coulter) to remove residual debris.

Envelope membranes migrated as a yellow/orange band at the interface between the stromal fraction and the 1.3 M sucrose cushion. The envelope fraction (~150 µL) was collected and pelleted at 150,000 × g for 1 h at 4 °C. The pellet was resuspended in 150 µL 0.33 MS buffer and re-pelleted at 150,000 × g for 30 min at 4 °C. The final envelope pellet was resuspended in 50 µL 0.33 MS buffer.

Thylakoid membranes accumulated at the interface between the 1.3 M and 1.8 M sucrose cushions. The thylakoid band (~200 µL) was collected, diluted 1:1 with 0.33 MS buffer, and pelleted at 15,000 × g for 15 min at 4 °C in a tabletop centrifuge.

Protein concentrations were determined by BCA assay using 5 µL aliquots from each fraction. Fractions were mixed with 5× SDS loading buffer (final 1×; ¼ volume added), incubated for 5 min at 95 °C, and analyzed by SDS–PAGE followed by immunoblotting. Compartment-specific marker proteins were used to assess fraction purity (see Results). Details of loading buffer composition, BCA assay conditions, and immunoblotting procedures are provided in the corresponding sections above.

Membrane subfractionation (*Synechocystis sp.* PCC 6803)

FLAG-VIA1 cells were grown in 500 mL of BG11 medium supplemented with Fe and glucose for 6 weeks. Cells were harvested by centrifugation at 12,000 rpm (18,514 x g) for 3 min and

resuspended in STN1 buffer (30 mM Tricine pH8, 0.4 M sucrose, 15 mM NaCl). DNase (5 µL) was added, and cells were lysed by passage through a French press (Constant systems CF1) at 30,000 psi for three cycles. The lysate was centrifuged at 12,000 rpm (18,514 x g) for 5 min to remove cellular debris, the supernatant was termed the whole cell lysate fraction (WCL). The WCL was subjected to ultracentrifugation at 45,000 rpm (208,000 x g) for 3 h at 4 °C. The supernatant was collected as the S1 fraction (the volume of this fraction was the reference volume for all washes and resuspension step). The S1 fraction was subjected to three additional ultracentrifugation steps at 45,000 rpm (208,000 x g) for 3 h at 4 °C to yield the final soluble fraction (Sol). The membrane pellet of the first ultracentrifugation step was resuspended in 0.75 volume of high-salt STN1 buffer (150 mM NaCl) and ultracentrifuged again at 45,000 rpm (208,000 x g) for 3 h at 4 °C. The pellet was resuspended again in 0.75 volume STN1 buffer to generate the membrane (Mem) fraction.

Co-Immunoprecipitation (*C. reinhardtii*)

A 5 mL preculture of *C. reinhardtii* WT cells (CrRS166) was grown in Tris–Acetate–Phosphate (TAP) medium at 22 °C under standard light conditions until a density of 2–6 × 10^6 cells mL^{-1} was reached. Cultures were sequentially expanded to 50 mL and then 150 mL to reach a final density of 2-6 × 10^6 cells mL^{-1}, at which point cells were harvested by centrifugation (3,000 × g, 5 min, 4 °C). The pellet was resuspended in a 1:1 (v/v) ratio of cell pellet to ice-cold 2× IP buffer (100 mM HEPES, 100 mM potassium acetate, 4 mM magnesium acetate, 2 mM calcium chloride, 400 mM sorbitol) supplemented immediately before use with EDTA-free protease inhibitors (Roche). Samples were snap-frozen in liquid nitrogen.

Cells were lysed by five freeze–thaw cycles (alternating thawing at room temperature and snap-freezing in liquid nitrogen). For solubilization, 300 µL lysate was mixed with 300 µL ice-cold IP buffer and 66 µL of 10% (w/v) digitonin (EMD Millipore) to achieve a final digitonin concentration of 1% (w/v). Samples were incubated on a rotating wheel (1 h, 4 °C) and clarified by centrifugation (21,300 × g, 30 min, 4 °C).

For immunoprecipitation, 25 µL Protein A Dynabeads (Thermo Fisher) were used per sample. Beads were washed four times with 1 mL IP buffer, resuspended in the original volume, and incubated with 4 µL of 1 mg mL^{-1} anti-VIPP1 antibody per sample (45 min, 20 °C, rotation). As negative controls, lysates were incubated with non-antibody-coupled beads.

After magnetic separation and one wash with IP buffer, beads were washed twice with IP buffer containing 0.1% (w/v) digitonin (5 min, 4 °C). Subsequently, 250 µL clarified lysate was added to the antibody-bound beads and incubated (3 h, 4 °C, rotation). Beads were then washed four times with IP buffer + 0.1% digitonin (first two washes 5 min; final two washes 10 min; all at 4 °C).

Elution was performed under denaturing conditions by resuspending beads in 60 µL 2× denaturing lysis buffer (100 mM Tris-HCl pH 8.0, 600 mM NaCl, 4% SDS, 20 mM EDTA) and incubating at 70 °C for 15 min (850 rpm). After magnetic separation, the supernatant was collected and stored at –20 °C.

For mass spectrometry preparation, 20 µL eluate was mixed with 5 µL 5× SDS loading buffer (250 mM Tris-HCl pH 6.8, 5% SDS, 0.025% bromophenol blue, 25% glycerol) supplemented with 5% (v/v) β-mercaptoethanol, incubated 5 min at 95 °C, separated by SDS-PAGE, and stained with colloidal Coomassie (MBS, VBCF). Gel lanes were excised, placed in 1% acetic acid, and stored at 4 °C until processing.

The same procedure was used for FLAG co-immunoprecipitation in *C. reinhardtii*, except that Magnetic Anti-FLAG M2 beads (Sigma-Aldrich) were used (25 µL per sample), and no antibody coupling step was required. The elution step was carried out under native conditions: beads were resuspended in 35 uL of 2X IP buffer supplemented with 10% digitonin and 1 µg/uL of 3XFLAG peptide (Peptide Facility, VBC), and incubated at 4 °C for 30 min in a thermomixer at 850 rpm. The supernatant was then separated and stored at -20 °C. This elution step was repeated 3 times, and the three elutions were pooled.

Co-Immunoprecipitation (*Synechocystis sp.* pcc 6803)

Synechocystis cells expressing VIA1 with an endogenous C-terminal FLAG tag were grown on selective BG11 agar plates for two weeks under continuous white light (~8 µmol photons m^{-2} s^{-1}). Colonies were inoculated into 50 mL BG11 and grown for two weeks, then expanded to 500 mL BG11 for two additional weeks. Cultures were further scaled to 10 L BG11 and grown for one week. For light adaptation, 500 mL cultures were exposed to 20 µmol photons m^{-2} s^{-1} for two weeks prior to harvest. Cells were collected by centrifugation and resuspended in an equal volume of ice-cold 2× IP buffer (see previous paragraph). The suspension was added dropwise into liquid nitrogen to generate frozen pellets, which were homogenized using a pre-chilled cryo-mill (Retsch; 5 cycles of 3 min at 20 s^{-1}, with 2 min cooling intervals in liquid nitrogen). The powdered material was thawed on ice and further homogenized by 30 strokes in a Kontes Dounce homogenizer.

For solubilization, 300 µL lysate was mixed with 300 µL IP buffer and 66 µL 10% digitonin (final concentration 1%), incubated 1 h at 4 °C, and clarified by centrifugation (21,300 × g, 30 min, 4 °C). From this point on, FLAG immunoprecipitation was performed as described in the previous paragraph.

Processing of Mass-Spectrometry samples (in-gel digest)

Coomassie-stained gel bands were cut to 2-3 mm pieces, transferred to 0.6 ml tubes and incubated with different solutions by shaking for 10 minutes at room temperature followed by removal of the supernatant as follows: Gel pieces were washed with 200 µl 100 mM ammonium bicarbonate (ABC), destained by two repeated rounds of shrinking in 200 µl 50% ACN in 50mM ABC and reswelling in 200 µl 100 mM ABC. Gel pieces were shrunk with 100 ul ACN before being reduced with 100 µl of 10 mM Dithiothreitol in 100 mM ABC by incubation at 57 °C for 30 min and alkylated with 100 µl of 20 mM Iodoacetamide in 100 mM ABC by incubation at RT for 30 min.

Wash steps were repeated as described for destaining, and gel pieces were shortly dried in a speed-vac after the final shrinking step. Gel pieces were soaked in 12.5ng/ul Trypsin in ABC for

5min at 4 °C. Excess solution was removed, ABC was added to cover the pieces, and samples were kept overnight at 37 °C. The supernatant containing tryptic peptides was transferred to a fresh tube, and gel pieces were extracted by the addition of 20 µL 5% formic acid and sonication for 10 min in a cooled ultrasonic bath. This step was performed twice. All supernatants were unified. A similar aliquot of each digest was analysed by LC-MS/MS.

NanoLC-MS/MS Analysis

The nano HPLC system (Vanquish Neo UHPLC-System, Thermo Scientific) was coupled to an Orbitrap Exploris 480 mass spectrometer equipped with a FAIMS pro interface or to an Orbitrap Astral mass spectrometer, both equipped with a Nanospray Flex ion source (Thermo Scientific). Peptides were loaded onto a trap column (PepMap Acclaim C18, 5 mm × 300 µm ID, 5 µm particle size, 100 Å pore size, Thermo Scientific) at a flow rate of 25 µl/min using 0.1% TFA as mobile phase. After 5 minutes, the trap column was switched in line with the analytical column (PepMap Acclaim C18, 500 mm × 75 µm ID, 2 µm particles, 100 Å, Thermo Scientific operated at 30 °C or Aurora Ultimate C18 25cm × 75 µm ID, 1.7 µm particles, 120 Å, with integrated emitter, Ionopticks operated at 50 °C). Peptides were eluted using a flow rate of 230 or 300 nl/min, starting with the mobile phases 98% A (0.1% formic acid in water) and 2% B (80% acetonitrile, 0.1% formic acid) and linearly increasing to 35% B over the next 60 or 120 minutes respectively, followed by an increase to 95% B in 1.7 min, a 4-min hold at 95% B, and re-equilibration with 2% B for three column volumes (equilibration factor of 3.0).

The Orbitrap Exploris 480 mass spectrometer was operated in data-dependent mode, performing a full scan (m/z range 350-1200, resolution 60,000, norm. AGC target 300%) at 3 different compensation voltages (CV -45, -60, -75), followed each by MS/MS scans of the most abundant ions for a cycle time of 0.9 seconds per CV. MS/MS spectra were acquired using HCD collision energy of 30, isolation width of 1.2 m/z, orbitrap resolution of 30,000, norm. AGC target 200%, minimum intensity of 25,000 and maximum injection time of 100 ms. Precursor ions selected for fragmentation (include charge state 2-6) were excluded for 45 s. The monoisotopic precursor selection filter and exclude isotopes feature were enabled.

The Orbitrap Astral was operated in data-independent mode, performing a full scan in the Orbitrap every 0.6 s (m/z range 380–980 m/z; resolution 240,000; AGC target 1,000,000, maximum injection time 5 ms). MS/MS spectra were acquired in the Astral analyser by isolating 4 Da windows across 380–880 m/z (resulting in 124 scan events per cycle) and fragmenting precursor ions with normalized HCD collision energy of 25% with a maximum injection time of 5 ms or until an AGC target of 30,000 was reached. Fragment ions ranging from 150–2000 m/z were acquired.

Data Processing MS protocol

Exploris

For peptide identification, the RAW-files were loaded into Proteome Discoverer (version 2.5.0.400, Thermo Scientific). All MS/MS spectra were searched using MSAmanda version

2.0.0.19924 (Dorfer V. et al., J. Proteome Res. 2014 Aug 1;13(8):3679-84). The peptide and fragment mass tolerance was set to ±10 ppm, the maximum number of missed cleavages was set to 2, using tryptic enzymatic specificity without proline restriction. The RAW-files were searched against the *C.reinhardtii* v6.1 Phytozome database (30,357 sequences; 24,276,980 residues), supplemented with common contaminants using the following modifications: Carbamidomethylation of cysteine was set as fixed modification and oxidation of methionine, deamidation of asparagine and glutamine, phosphorylation on serine, threonine and tyrosine, ubiquitylation residue on lysine, glutamine to pyro-glutamate conversion at peptide N-terminal glutamine and acetylation on the protein N-terminus were set as variable modifications. The localization of the post-translational modification sites within the peptides was performed with the tool ptmRS, based on the tool phosphoRS[17].

The result was filtered to 1 % FDR on PSM and protein level using the Percolator algorithm[18] integrated in Proteome Discoverer. Additionally, an Amanda score cut-off of at least 150 was applied. Proteins were filtered to be identified by a minimum of 2 PSMs in at least 1 sample.

Protein areas were computed in IMP-apQuant[19] by summing up unique and razor peptides. Resulting protein areas were normalized using iBAQ[20]. Match-between-runs (MBR) was applied for peptides with high confident peak area that were identified by MS/MS spectra in at least one run. Proteins were filtered to be identified by a minimum of 3 quantified peptides.

Astral

Astral DIA data were analyzed in Spectronaut 20.1[20] (Biognosys). Trypsin/P was specified as a proteolytic enzyme, and up to 2 missed cleavages were allowed in the Pulsar directDIA+ search. Dynamic mass tolerance was applied for calibration and the main search. The RAW files were searched against the *C. reinhardtii* v6.1 Phytozome database (30,357 sequences; 24,276,980 residues), supplemented with common contaminants using the following modifications: Carbamidomethylation of cysteine was searched as fixed modification, whereas oxidation of methionine and acetylation at protein N-termini were defined as variable modifications. Peptides with a length between 7 and 52 amino acids were considered, and results were filtered using Spectronaut default filtering criteria (Precursor Qvalue<0.01, Precursor PEP<0.2, Protein Qvalue <0.01 per Experiment and <0.05 per Run, Protein PEP<0.75). Quantification was performed as specified in Biognosys BGS Factory Default settings, grouping peptides by stripped sequence and performing protein inference using IDPicker. Cross-run normalization in Spectronaut was deactivated due to subsequent mode normalization.

Spectronaut results were exported using Pivot reports on the protein and peptide level and converted to Microsoft Excel files using our in-house software MS2Go (https://ms.imp.ac.at/?action=ms2go). For DIA data MS2Go utilizes the Python library msReport (developed at the Max Perutz Labs Proteomics Facility) for data processing. Abundances were normalized by the mode of protein ratios in msReport and missing values were imputed with values obtained from a log-normal distribution with a mean of 100. To compensate for different protein lengths, protein quantification was then normalized using iBAQ[20]. Statistical significance of differentially expressed proteins was determined using limma[21].

The mass spectrometry proteomics data have been deposited to the ProteomeXchange Consortium via the PRIDE[22] partner repository with the dataset identifier PXD072529.

Molecular Cloning (*C. reinhardtii*)

The plasmid pRAM315 was generated via In-Fusion cloning (Takara) using pRAM254 as the backbone; pRAM254 contained the *VIA1* promoter (1,500 bp upstream of the 5'UTR), the 5'UTR, the full *VIA1* coding sequence (CDS), a NeonGreen sequence, and a 3×FLAG epitope in frame to the C-terminus of *VIA* CDS. Two PCRs were performed to amplify the genomic locus of *VIA1* from genomic DNA, with the primers oRS41-42 (TM 65 °C, extension time 4') and oRS43-44 (TM 63 °C, extension time 3' 30''). To clone pRAM315, oRS401-402 were used to amplify the 3XFLAG epitope, oRS403-404 to amplify the 3'UTR from genomic DNA (TM 60 °C, extension time 1'), and oRS405-406 were used to linearize the backbone (pRAM254) and remove the NeonGreen sequence. All remaining plasmids were assembled via Golden Gate cloning, following the strategy and toolkit developed in[23.] Briefly, pCSB192 was used as the backbone to insert the coding sequences of *mutVIA1*, *SynVIA1*, and *AtVIA1*, generating level-0 constructs. In the subsequent reaction, the 16S promoter and RbcL terminator/3'UTR were added using plasmids pStA0_16S and pStA0_TrbcL, respectively, with pGT402 as the backbone, yielding level-1 plasmids. In the final step, right and left homology arms targeting the *psbH* locus were incorporated from pHT150 and pHT146, respectively, along with the spectinomycin resistance cassette (*aadA*) from pCSB176. The final level-2 and final constructs were assembled using pCSB192 as the backbone. All plasmids were sequence-verified prior to transformation into Chlamydomonas. A complete list of plasmids and primers used in this study can be found in Tables S3 and S5, respectively.

Molecular Cloning (*Synechocystis sp.* pcc 6803)

A plasmid containing the *SynVIA1* gene fused to a C-terminal triple FLAG tag and marked with a chloramphenicol resistance cassette (Cm^R) was assembled from five PCR-amplified fragments. The downstream fragment was amplified from Synechocystis genomic DNA using primer pair 446/447. The *SynVIA1* gene with 1000 bp of its upstream region was amplified using primers 442/443 and Synechocystis genomic DNA as the template. The plasmid backbone containing the ColE1 origin of replication was amplified from pJET1.2 using primers 448/441. The Cm^R cassette was amplified from pACYC184 (New England Biolab) using primers 444/445. All fragments were assembled using the NEBuilder® HiFi DNA Assembly Master Mix.

The SynVIA1 deletion mutant was generated by replacing the *SynVIA1* coding region with a Kanamycin resistance cassette (Kan^R) amplified from pUC57-Kan^R (GenScript). The upstream and downstream flanking sequences were amplified from Synechocystis genomic DNA using primer pairs 442/449 and 446/447, respectively. The Kan^R cassette was amplified using primers 450/445, and the backbone fragment containing the ColE1 origin of replication was amplified from pJET1.2 (REF) with primers 448/441. The four fragments were assembled using the NEBuilder® HiFi DNA Assembly Master Mix. All plasmids were sequence-verified prior to transformation into

Synechocystis. Complete deletion of *SynVIA1* was confirmed by PCR using primers 450/445, restriction enzyme digestion with NheI, and sequencing.

Nuclear transformation (*C. reinhardtii*)

Nuclear transformation was performed by electroporation as described in ref. 18. Briefly, *via1-1* cells were grown in TAP medium to mid-log phase ($2–4 \times 10^6$ cells mL^{-1}), harvested by centrifugation (800 x g for 5’), and washed twice with 5 mL CHES buffer (10 mM N-cyclohexyl-2-aminoethanesulfonic acid (CHES), pH 9.25, 40 mM sucrose, 10 mM sorbitol). Cells were resuspended in CHES buffer to a final density of 8×10^8 cells mL^{-1}.

For each transformation, 1 µg linearized pRAM315 was mixed with 0.5 µL salmon sperm DNA (10 mg mL^{-1}; Invitrogen 15-632-011) and added to 125 µL of washed cells. The mixture was transferred to 2-mm gap electroporation cuvettes and electroporated using a NEPA21 Type II square-pulse electroporator (ref. 19). The electroporation program consisted of two poring pulses (250 V and 150 V, 8 ms each), followed by five transfer pulses (50 ms each) starting at 20 V with a 40% decay (20, 12, 7.2, 4.3, and 2.6 V).

Immediately after electroporation, cells were recovered in 8 mL TAP supplemented with 40 mM sucrose and incubated overnight at room temperature under low light with gentle shaking. Cells were then harvested (800 x g for 5’), resuspended in 1 mL TAP, and plated on TAP agar containing 20 µg mL^{-1} hygromycin. Hygromycin-resistant colonies were screened for transgene expression by anti-FLAG immunoblotting.

Chloroplast transformation (*C. reinhardtii*)

Chloroplast biolistic transformation of the *via1-1* strain was performed using a helium-driven particle gun based on the designs described in[24,25], and following the protocol in[25]. Cells were grown to ~4×10^6 cells mL^{-1}, harvested by centrifugation (800 x g for 5’), and resuspended in TAP to a final density of 1×10^8 cells mL^{-1}. 300 µL of concentrated cells was spread onto TAP agar plates containing 100 µg mL^{-1} spectinomycin (Sigma-Aldrich) and air-dried in a laminar-flow hood for ~15 min. Plates were bombarded with 550-nm gold particles (Seashell Technology) coated with 1 µg plasmid DNA (pRAM321, pRAM537, or pRAM324, Table S3). After bombardment, plates were incubated under continuous light (80 µmol photons m^{-2} s^{-1}) for ~10 days until colonies appeared. Transformants were restreaked at least four times on selective medium to achieve homoplasmy, and transgene expression was verified by RT-qPCR.

Transformation (*Synechocystis sp.* pcc 6803*)*

Wild-type *Synechocystis sp.* pcc 6803 grown in BG11 liquid medium supplemented with 6 µg/ml Ferric ammonium citrate and 5mM glucose under continuous white light (~8 µmol photons m^{-2} sec^{-1}). Once saturated, cells were diluted 10x the day before nuclear transformation. On the day of transformation, cells were harvested when the culture reached an optical density at 730 nm of 0.2–0.6. For each transformation, 5 mL of culture was collected by centrifugation at 3000 x g for 5 minutes. The cell pellet was resuspended in 1 mL of BG11 medium supplemented with iron and

transferred to a microcentrifuge tube. Cells were centrifuged again at 2000 x g for 2 minutes and resuspended in 1 mL of BG11 medium. Aliquots of 100 µL were prepared for each transformation. For each transformation, at least 200 ng of plasmid DNA was added to the cell suspension. The transformation mixtures were incubated in the dark for 5 hours. Following incubation, cells were plated on selective BG11 agar plates containing a limiting concentration of the appropriate antibiotic. Colonies were typically observed after 3–4 weeks.

References

1. Perlaza, K. *et al.* The MARS1 kinase confers photoprotection through signaling in the chloroplast unfolded protein response. *Elife* 8, (2019).

2. Werner, R. & Mergenhagen, D. Mating Type Determination of Chlamydomonas reinhardtii by PCR. *Plant Mol. Biol. Report.* 16, 295–299 (1998).

3. Buchfink, B., Reuter, K. & Drost, H.-G. Sensitive protein alignments at tree-of-life scale using DIAMOND. *Nat. Methods* 18, 366–368 (2021).

4. Richter, D. J. *et al.* EukProt: A database of genome-scale predicted proteins across the diversity of eukaryotes. *Peer Community Journal* 2, e56 (2022).

5. The UniProt Consortium: UniProt: a worldwide hub of protein knowledge. *Nucleic Acids Res.* 47, D506–D515 (2019).

6. Katoh, K. & Standley, D. M. MAFFT Multiple Sequence Alignment Software Version 7: Improvements in Performance and Usability. *Mol. Biol. Evol.* 30, 772–780 (2013).

7. Capella-Gutiérrez, S., Silla-Martínez, J. M. & Gabaldón, T. trimAl: a tool for automated alignment trimming in large-scale phylogenetic analyses. *Bioinformatics* 25, 1972–1973 (2009).

8. Nguyen, L.-T., Schmidt, H. A., von Haeseler, A. & Minh, B. Q. IQ-TREE: A Fast and Effective Stochastic Algorithm for Estimating Maximum-Likelihood Phylogenies. *Mol. Biol. Evol.* 32, 268–274 (2015).

9. Guindon, S. *et al.* New Algorithms and Methods to Estimate Maximum-Likelihood Phylogenies: Assessing the Performance of PhyML 3.0. *Syst. Biol.* 59, 307–321 (2010).

10. Mistry, J., Finn, R. D., Eddy, S. R., Bateman, A. & Punta, M. Challenges in homology search: HMMER3 and convergent evolution of coiled-coil regions. *Nucleic Acids Res.* 41, e121–e121 (2013).

11. Kalyaanamoorthy, S., Minh, B. Q., Wong, T. K. F., von Haeseler, A. & Jermiin, L. S. ModelFinder: fast model selection for accurate phylogenetic estimates. *Nat. Methods* 14, 587–589 (2017).

12. Letunic, I. & Bork, P. Interactive Tree of Life (iTOL) v6: recent updates to the phylogenetic tree display and annotation tool. *Nucleic Acids Res.* 52, W78–W82 (2024).

13. Li, H. Protein-to-genome alignment with miniprot. *Bioinformatics* 39, (2023).

14. Demmig-Adams B, Garab G., Adams W. III, Govindjee (Eds.) Non-Photochemical Quenching and Energy Dissipation in Plants, Algae and Cyanobacteria. vol. 40 *Springer Netherlands, Dordrecht* https://doi.org/10.1007/978-94-017-9032-1 (2014).

15. Klughammer, C. & Ulrich, S. Complementary PS II quantum yields calculated from simple fluorescence parameters measured by PAM fluorometry and the Saturation Pulse method. *PAM application notes* 201247 (2008).

16. Sun, Y. & Zerges, W. Translational regulation in chloroplasts for development and homeostasis. *Biochimica et Biophysica Acta (BBA) - Bioenergetics* 1847, 809–820 (2015).

17. Taus, T. *et al.* Universal and Confident Phosphorylation Site Localization Using phosphoRS. *J. Proteome Res.* 10, 5354–5362 (2011).

18. Käll, L., Canterbury, J. D., Weston, J., Noble, W. S. & MacCoss, M. J. Semi-supervised learning for peptide identification from shotgun proteomics datasets. *Nat. Methods* 4, 923–925 (2007).

19. Doblmann, J. *et al.* apQuant: Accurate Label-Free Quantification by Quality Filtering. *J. Proteome Res.* acs.jproteome.8b00113 (2018) doi:10.1021/acs.jproteome.8b00113.

20. Schwanhäusser, B. *et al.* Global quantification of mammalian gene expression control. *Nature* 473, 337–342 (2011).

21. Smyth, G. K. Linear Models and Empirical Bayes Methods for Assessing Differential Expression in Microarray Experiments. *Stat. Appl. Genet. Mol. Biol.* 3, 1–25 (2004).

22. Perez-Riverol, Y. *et al.* The PRIDE database resources in 2022: a hub for mass spectrometry-based proteomics evidences. *Nucleic Acids Res.* 50, D543–D552 (2022).

23. Taylor, G. M., Mordaka, P. M. & Heap, J. T. Start-Stop Assembly: a functionally scarless DNA assembly system optimized for metabolic engineering. *Nucleic Acids Res.* 47, e17–e17 (2019).

24. Finer, J. J., Finer, K. R. & Ponappa, T. Particle Bombardment Mediated Transformation. in 59–80 (2000). doi:10.1007/978-3-642-60234-4_3.

25. Klein, U. & Bogorad, L. Biolistic-Based Analysis of Chloroplast Gene Expression. in *Gene Transfer to Plants* 162–169 (Springer Berlin Heidelberg, Berlin, Heidelberg, 1995). doi:10.1007/978-3-642-79247-2_20.
